# Sunitinib enhances cuproptosis induced by copper ionophores via ROS-ATF3-SLC31A1 axis in thyroid Carcinoma

**DOI:** 10.1038/s41419-026-08878-9

**Published:** 2026-06-01

**Authors:** Dan Zhao, Yunjun Wang, Qing Guan, Weibo Xu, Zhou Yang, Xiangze Li, Tian Liao, Yu Wang, Xiang Zhou, Jun Xiang

**Affiliations:** 1 Department of Head and Neck Surgery, Fudan University Shanghai Cancer Center, Shanghai, China; 2 Department of Oncology, Shanghai Medical College, Fudan University, Shanghai, China; 3 Fudan University Shanghai Cancer Center and Institutes of Biomedical Sciences, Fudan University, Shanghai, China

**Keywords:** Thyroid cancer, Targeted therapies

## Abstract

The clinical management of advanced thyroid carcinoma presents substantial therapeutic challenges due to non-curative surgical outcomes and resistance to both radioiodine therapy and conventional chemotherapeutic agents. Emerging evidence implicates copper dysregulation in thyroid cancer pathogenesis, yet monotherapy with copper ionophores demonstrates constrained efficacy due to tumor-intrinsic adaptive resistance mechanisms. In this study, we investigated the therapeutic potential of combining Sunitinib with copper ionophore Elesclomol as a novel strategy against thyroid carcinoma. Mechanically, we identified that Sunitinib treatment induces ROS accumulation, activating the stress-responsive transcription factor ATF3, which directly binds and transactivates the copper transporter gene SLC31A1. Clinically, TCGA analysis reveals SLC31A1 as a vulnerability in advanced thyroid cancer, with expression significantly downregulated in metastatic lesions, a deficit rescued by Sunitinib. Overall, these results demonstrate that Sunitinib and copper ionophores can induce synergistic cytotoxic effect, shedding light on therapeutic strategy for advanced thyroid carcinomas.

## Introduction

Tyrosine kinase inhibitors (TKIs) are currently available as critical therapeutic options for advanced thyroid malignancies, including radioiodine-refractory differentiated thyroid carcinoma, metastatic medullary thyroid carcinoma (MTC) and anaplastic thyroid carcinoma (ATC) [1–3]. However, acquired resistance frequently develops via incompletely characterized metabolic reprogramming, while treatment-related toxicities constrain their applicability in vulnerable subpopulations [4]. These limitations underscore the critical unmet need for innovative therapeutic approaches. Consequently, growing researches investigate TKI-based combination regimens with complementary systemic agents [5].

Accumulating evidence implicates a complex relationship between dysregulated copper homeostasis and thyroid malignancies pathogenesis [6]. Elevated serum copper concentrations and augmented cellular copper import observed in patients with thyroid cancer indicate that copper dysregulation may represent a cancer hallmark [7, 8]. The identification of cuproptosis, a copper-dependent regulated cell death mechanism, has stimulated investigation of novel oncologic therapeutics [9–11]. Previous studies have shown that malignancies with high copper uptake and high mitochondrial metabolism exhibit high sensitivity to copper ionophores (e.g., elesclomol, disulfiram) [12]. Cuproptosis induction, therefore, constitutes a promising therapeutic paradigm for thyroid carcinoma. However, monotherapy efficacy remains constrained. While this pathway can be pharmacologically induced by upregulating SLC31A1 (CTR1) or administering copper ionophores [11, 13], its therapeutic efficacy is largely compromised by tumor-intrinsic resistance mechanisms. These include glutathione-mediated redox buffering, ATP7A/B-driven copper efflux, and metabolic plasticity, all of which collectively limit the cytotoxic accumulation of Cu²⁺[14]. Overcoming these resistance barriers to achieve tumor-selective copper overload represents a critical translational objective for optimizing cuproptosis-based therapies [15].

Vascular endothelial growth factor receptor 2 (VEGFR2, also known as KDR/FLK1) represents a principal molecular target for TKIs [16]. While genomically guided TKIs (e.g., BRAF-directed agents) have advanced precision oncology, the majority of patients who lack these actionable mutations remain candidates for antiangiogenic TKIs that inhibit the VEGFR pathway [17]. Previous research has delineated molecular crosstalk between angiogenic pathways and copper homeostasis [18–20]. For instance, Das A et al. demonstrated that the copper influx transporter SLC31A1 not only mediates copper entry but also undergoes physical co-internalization with VEGFR2, thereby sustaining pro-angiogenic signaling [21]. This finding establishes a direct mechanistic link between copper metabolism and angiogenic pathways.

Building upon established mechanistic insights, we investigated the therapeutic potential of combining Sunitinib with copper ionophores as a novel strategy against thyroid carcinoma. By elucidating the mechanistic basis and validating efficacy across multiple preclinical models, this study aims to pave the way for a promising new approach to combat advanced thyroid cancer.

## Materials and methods

### Cell culture and reagents

Human thyroid cancer cell lines including B-CPAP (RRID: CVCL_0153), TPC-1 (RRID: CVCL_6298), K1 (RRID: CVCL_2537), FTC-133 (RRID: CVCL_1219), MZ (RRID: CVCL_ZC26), TT (RRID: CVCL_1774), C-643 (RRID: CVCL_5969), BHT-101 (RRID: CVCL_1085), KHM-5M (RRID: CVCL_2975), and SW1736 (RRID: CVCL_3883) and HEK293T (RRID: CVCL_0063) cells were obtained from the American Type Culture Collection (ATCC). All cell lines underwent rigorous quality control, including authentication via short tandem repeat (STR) profiling, confirming consistency with ATCC reference profiles and contamination free. B-CPAP, TPC-1, C-643, BHT-101, KHM-5M, and SW1736 cells were cultured in RPMI-1640 medium supplemented with 10% fetal bovine serum (FBS; Gibco), K1 and TT cells were maintained in Ham’s F-12K (Kaighn’s) medium with 10% FBS, MZ cells were maintained in Dulbecco’s Modified Eagle Medium (DMEM) with 20% FBS, HEK293T cells were cultured in DMEM medium supplemented with 10% FBS and FTC-133 cells were maintained in DMEM/F12K with 10% FBS. All media contained 100 U/mL streptomycin and 100 μg/mL penicillin. The cuproptosis inducer elesclomol (ES) and inhibitor tetrathiomolybdate (TTM) were purchased from MedChemExpress. Additional inhibitors included the ferroptosis inhibitor ferrostatin-1 (Fer-1), necroptosis inhibitor necrostatin-1s (Nec-1s), and apoptosis inhibitor Z-VAD-FMK, all sourced from MedChemExpress.

### Bioinformatics analysis

The relative clinical data of thyroid carcinoma (THCA) transcriptome profiles were obtained from The Cancer Genome Atlas (TCGA, https://portal.gdc.cancer.gov), TCGA database were applied to validate the differential expression of SLC31A1 and ATF3 in tumor tissues and normal tissues. The edge R package of R (V4.3.2, http://www.bioconductor.org) was used to analysis data from the online database, RNA sequencing and CUT&Tag sequencing.

### Immunohistochemistry staining

Immunohistochemistry (IHC) staining was performed using antibodies against SLC31A1 (Cat# 67221-1-Ig, Proteintech) and Ki-67 (Cat# 27309-1-AP, Proteintech) to evaluate protein expression in human and xenograft tissues. Staining intensity and positivity were scored as follows: Positivity: 0 (no positive cells), 1 ( < 10%), 2 (11–50%), 3 (51–80%), 4 ( > 80%). Intensity: 0 (no staining), 1 (pale yellow), 2 (yellow), 3 (brown). The final score was calculated by multiplying positivity and intensity values.

### Plasmid and lentivirus production

To knock down SLC31A1 and ATF3 expression, shRNA sequences targeting SLC31A1 and ATF3 were cloned into the pCDH-U6-shRNA-EF1-GFP-puro lentiviral vector. For ATF3 overexpression, Flag-tagged ATF3 was inserted into the pcDNA3.1-MCS-EGFP-puro vector. The siRNA sequences and shRNA sequences are listed in Table S1 and Table S2 in Supporting Information.

### Immunoblotting

Protein extraction was performed with ice-cold RIPA lysis buffer, supplemented with phosphatase and protease inhibitor cocktails (1:100 dilution). Lysates were centrifuged at 12,000 × *g* for 15 min at 4 °C to obtain clarified supernatants. Protein concentrations were determined by BCA assay, and equal amounts (20 μg) were resolved by SDS-PAGE prior to transfer to PVDF membranes for immunoblotting analysis. Primary antibodies included: ATF3 (1:1000; #18665S, Cell Signaling Technology), SLC31A1 (1:1000; #67221-1-Ig, Proteintech), GAPDH (1:10,000; #10494-1-AP, Proteintech), FDX1 (1:1000; #12592-1-AP, Proteintech), LIAS (1:1000; #11577-1-AP, Proteintech), DLAT (1:1000; #13426-1-AP, Proteintech), Lipoic Acid (1:1000; sc-101354, Santa Cruz Biotechnology), MTCO2 (1:1000; #A11522, Abclonal), VDAC (1:10,000; #A27273PM, Abclonal), NDUFB8 (1:1000; #A20457, Abclonal), SDHB (1:1000; #A10821, Abclonal), UQCRC2 (1:1000; #A4181, Abclonal), and ATP5A1 (1:1000; #A11217, Abclonal). Protein band intensities were quantified using Fiji ImageJ software.

### Quantitative reverse transcription PCR (RT-qPCR)

Total RNA was isolated from huma PTC cells using the FastPure Cell/Tissue Total RNA Isolation Kit V2 (Vazyme Biotech). RNA purity and concentration were verified spectrophotometrically (A260/A280 ratio >1.8). Complementary DNA (cDNA) synthesis was performed with 1 μg total RNA using the PrimeScript RT Reagent Kit (TaKaRa Bio) under manufacturer-specified conditions. Quantitative real-time PCR (RT-qPCR) analysis was conducted using SYBR Green Master Mix (TaKaRa Bio) on a QuantStudio 6 Pro system. Relative gene expression was calculated via the 2^−ΔΔCt^ method with normalization to GAPDH endogenous control. Primer sequences are provided in Supplementary Table S3.

### Cell proliferation assays in vitro

For cell viability assessment, human papillary thyroid carcinoma (PTC) cells were plated in 96-well plates (5 × 10³ cells/well) and allowed to adhere for 24 h prior to treatment. Following intervention of 24 h, 10 μL CCK-8 reagent (Vazyme Biotech) was added per well. Plates were incubated at 37 °C in the dark for 3 h, after which absorbance was measured at 450 nm using a Bio-Rad iMark microplate reader. Dose-response curves were generated through nonlinear regression analysis (four-parameter logistic model) in GraphPad Prism v9.0. Half-maximal inhibitory concentrations (IC₅₀) were derived from the concentration required to reduce cell viability by 50% relative to vehicle-treated controls, with values representing mean ± SD of three independent experiments performed in technical triplicate.

### Flow cytometry analysis

Apoptosis quantification was performed using the Annexin V-PE/7-AAD and Annexin V-FITC/PI Apoptosis Detection Kit (Yeasen Biotechnology) per manufacturer’s protocol. Briefly, treated cells were harvested, washed twice with ice-cold PBS, and resuspended in 1× binding buffer at 1 × 10⁶ cells/mL. Cell suspensions were dual-stained with 5 μL Annexin V-PE (Annexin V-FITC) and 10 μL 7-AAD (PI), followed by 15 min incubation at room temperature in darkness. Samples were analyzed within 1 h on a BD FACSCalibur flow cytometer (BD Biosciences) equipped with 488 nm excitation. Data represent three independent experiments with ≥10,000 events acquired per sample.

### Long-term clonogenic assays

For colony formation assays, cells were seeded in 24-well plates at a density of 200 cells/well (adjusted according to proliferation kinetics) and cultured for 10–14 days in medium containing specified compounds, with biweekly medium replacement. Following incubation, cells were fixed with 4% formaldehyde in PBS for 15 min and stained with 0.1% (w/v) crystal violet aqueous solution for 30 min at room temperature. Colonies were subsequently imaged and quantified.

### Cell migration assay in vitro

Cell migration was assessed using 8 μm pore polycarbonate transwell inserts (Corning). Briefly, for Transwell migration assays, cells were seeded into the upper chamber at a density of 5 × 10⁴ cells per well in 200 μL serum-free medium. Lower chambers contained 600 μL complete medium with 10% FBS (Gibco) as chemoattractant. Following 24 h incubation at 37 °C/5% CO₂, non-migrated cells were mechanically removed from the upper membrane surface using cotton-tipped applicators. Migrated cells on the lower membrane surface were fixed with 4% paraformaldehyde (15 min, RT), stained with 0.1% crystal violet (20 min), and extensively washed. Cells were quantified in five random fields per membrane at 200× magnification using a Nikon Eclipse Ti2 microscope. Data represent mean migrated cells/field ± SEM from three independent experiments (*n* = 3 replicates per condition).

### Animal models

According to established guidelines for preclinical animal studies, a group size of 5 or 6 is widely considered sufficient to detect a biologically meaningful difference when the effect size is expected to be large. Four-week-old female nude mice were obtained from Shanghai SLAC Laboratory Animal Co., Ltd. (Shanghai, China). B-CPAP cells (2 × 10⁶ cells/mouse) were subcutaneously inoculated into the right flank. Mice were randomized into four groups (*n* = 5/group): Vehicle control: DMSO (0.1 mL, intragastric administration [i.g.], 5×/week. Sunitinib monotherapy: 40 mg/kg Sunitinib (i.g., 5×/week). ES&Cu monotherapy: Elesclomol (30 mg/kg) + CuCl₂ (2 mg/kg, i.g., every other day). Combination therapy: Sunitinib + ES&Cu (dosing as above). Tumor volume was monitored weekly using calipers and calculated as V=length×width^2^×0.5=length×width^2^×0.5. At day 20 post-implantation, tumors were excised, fixed in 4% paraformaldehyde, and processed for immunohistochemistry (IHC). The animals were allocated to experimental groups based on body weight to ensure comparable starting conditions between groups. No formal randomization procedure was employed in this study. The investigators were not blinded to group allocation during the experiment or outcome assessment. All procedures were approved by the Committee on the Ethics of Animal Experiments of Fudan University Shanghai Cancer Center (FUSCC-IACUC-2024529).

### RNA sequencing analysis

Total RNA was isolated from PTC cells using TRIzol™ Reagent (Sigma-Aldrich) according to the manufacturer’s protocol. RNA integrity was verified by Bioanalyzer 2100 (Agilent Technologies; RIN ≥ 8.0). Stranded cDNA libraries were prepared with the TruSeq Stranded mRNA LT Kit (Illumina) and sequenced (2 × 150 bp paired-end) on an Illumina HiSeq 4000 platform targeting 40 million reads per sample. Raw sequencing data underwent quality control (FastQC v0.11.9), adapter trimming (Trimmomatic v0.39), and alignment to the GRCh38 reference genome (STAR v2.7.10a). Transcript abundance was quantified as fragments per kilobase per million mapped reads (FPKM) using Cufflinks v2.2.1.

### CUT&Tag analysis

Paired-end sequencing reads (150 bp) were aligned to the human reference genome GRCh38 (GENCODE v38) using Bowtie2 v2.4.2 with default parameters. Duplicate reads from PCR amplification artifacts were marked and removed using Picard Tools v2.27.4 MarkDuplicates. Normalized coverage tracks in BigWig format were generated with deepTools v3.5.1 bamCoverage (RPGC normalization, bin size = 10 bp). Significant enrichment peaks were identified using MACS2 v2.2.7.1 (*q* < 0.05, --broad flag for histone marks) and annotated to genomic features (promoters: ±2 kb from TSS; exons; intergenic regions) with HOMER v4.11 annotatePeaks.pl. Data visualization and quality validation were performed using Integrative Genomics Viewer v2.12.3.

### Chromatin immunoprecipitation (ChIP) assay

Briefly, 4 × 10⁶ thyroid cancer cells were crosslinked with 1% formaldehyde for 10 min at room temperature, followed by quenching with 0.125 M glycine. Cells were then washed twice with ice-cold PBS and collected by centrifugation. Isolated nuclei were digested with 0.5 μL Micrococcal Nuclease (per 4 × 10⁶ cells) at 37 °C for 20 min to fragment chromatin. Protein-DNA complexes were extracted and resuspended in SimpleChIP® Chromatin IP Buffers (Cell Signaling Technology, Danvers, MA, USA). Immunoprecipitation was carried out following a standard co-immunoprecipitation protocol. After extensive washing, bound DNA was eluted using MinElute Spin Columns (Qiagen, Hilden, Germany) and analyzed by quantitative real-time PCR (qRT-PCR) or next-generation sequencing. Primer sequences used in ChIP-qPCR are listed in Supplementary Table S3.

### Luciferase reporter assay

To investigate ATF3-mediated transcriptional regulation of SLC31A1, wild-type (WT) and mutant (MUT) SLC31A1 promoter fragments ( − 2000 to +250 relative to the transcription start site) were cloned into the pGL3-Basic vector (Promega). Cells were co-transfected with: pGL3-Basic-SLC31A1-WT + pcDNA3.1-ATF3, pGL3-Basic-SLC31A1-MUT + pcDNA3.1-ATF3 or corresponding empty vectors (controls). Transfections included the internal control vector pRL-TK (Promega) and utilized Lipofectamine™ 2000 (Invitrogen, Carlsbad, CA). Luciferase activity was measured 48 h post-transfection using the Dual-Luciferase Reporter Assay System (Beyotime) on a ZS/BK-L96C chemiluminescence analyzer. Data represent mean ± SEM of three biological replicates, each with technical triplicates.

### Total reactive oxygen species (ROS) Level Assay

Intracellular ROS generation were assessed using the 2’,7’-dichlorodihydrofluorescein diacetate (DCFH-DA; Yeasen Biotechnology). Cells were seeded in 6-well plates, treated for 24 h, washed with PBS, and incubated with 5 μM DCFH-DA in serum-free medium at 37 °C for 30 min in the dark. Fluorescence intensity was measured via flow cytometry (Beckman CytoFLEX) using FITC channel settings (Ex/Em: 488/525 nm).

### Drug combination analysis

The synergistic effects of Sunitinib and ES&Cul were evaluated using the Chou-Talalay method [22] via CompuSyn software (ComboSyn Inc.). Combination index (CI) values were calculated and plotted against the fraction affected (FA), where CI < 1 indicates synergy, CI = 1 denotes additivity, and CI > 1 reflects antagonism.

### Measurement of oxygen consumption rate (OCR)

OCR was assessed using the Seahorse XFe24 Flux Analyzer (Agilent Technologies) with the Mitochondrial Stress Test Kit (Seahorse Bioscience, Cat# 103015-100). Briefly, 2 × 10^5^ cells/well were seeded in a 96-well XF Seahorse plate and equilibrated in XF base medium (pH 7.4) at 37 °C. Sequential injections of glucose (10 mM), glutamine (1 mM), 2-deoxyglucose (2-DG, 50 mM), and oligomycin (1 μM) were performed according to manufacturer protocols. OCR was measured in real-time and analyzed using Seahorse XF24 software (v2.6.1).

### Statistical analysis

Statistical analyses were performed using GraphPad Prism v9.01, SPSS v24, and R v4.3.2. Continuous variables were analyzed using paired Student’s *t*-test. Correlations between gene expression levels were assessed using Pearson (parametric) or Spearman (non-parametric) coefficients. Data are presented as mean ± SEM from three independent experiments (each with duplicate technical replicates). Two-tailed *p*-values were interpreted as follows: *P < 0.05, **P < 0.01, ***P < 0.001.

## Results

### Copper ionophore induced cytotoxicity in thyroid cancer cells

**Fig. 1:**
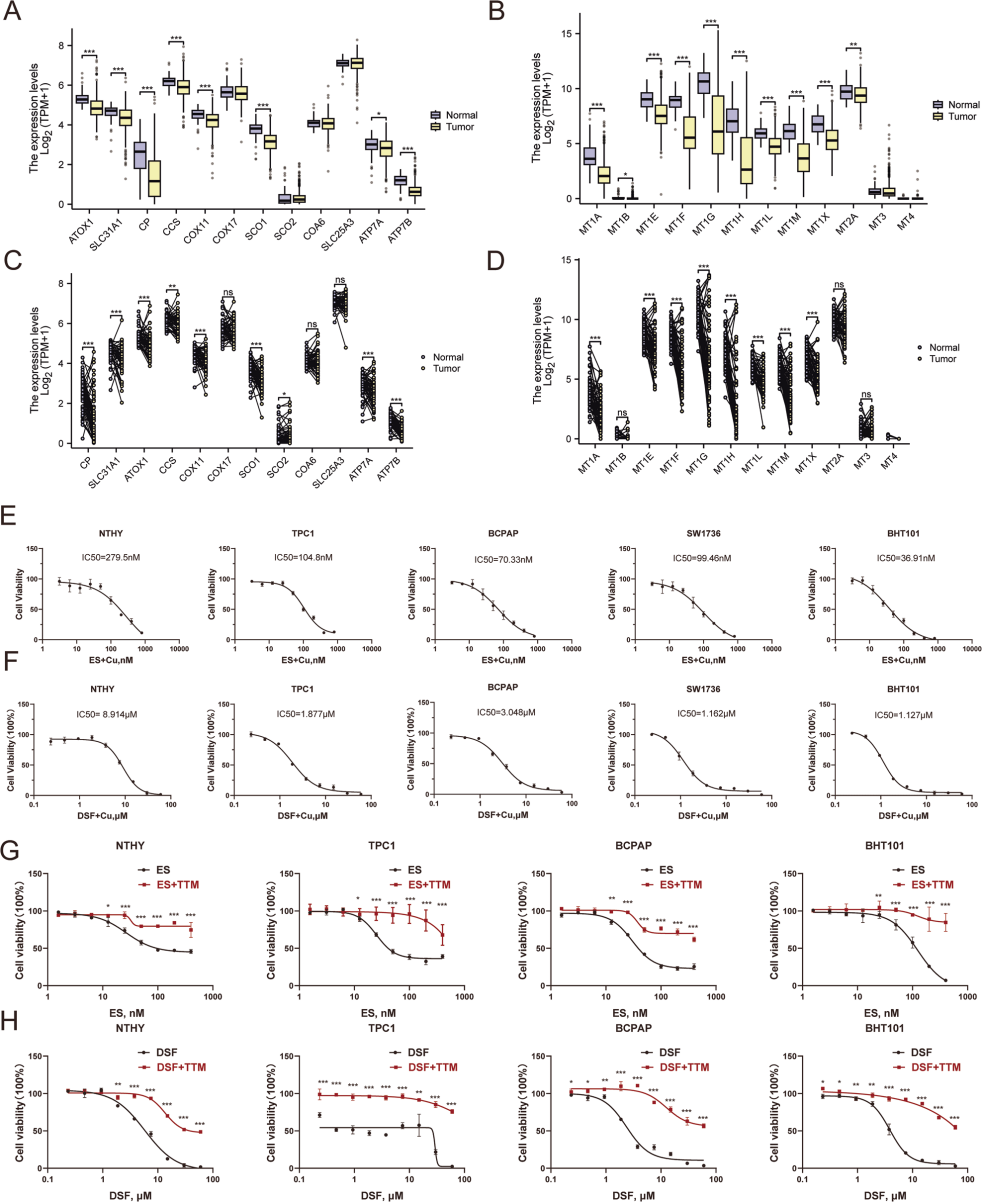
Cytotoxicity induced by copper ionophores in thyroid cancer cells. **A** Differential expression of 12 genes involved in copper homeostasis regulation between unmatched tumor (T) and adjacent normal (N) tissues in the TCGA_THCA dataset. **B** Differential expression of 12 metallothionein genes between unmatched tumor (T) and adjacent normal (N) tissues in the TCGA_THCA dataset. **C** Differential expression of 12 genes involved in copper homeostasis regulation between matched tumor (T) and adjacent normal (N) tissues in the TCGA_THCA dataset. **D** Differential expression of 12 metallothionein genes between matched tumor (T) and adjacent normal (N) tissues in the TCGA_THCA dataset. **E** Viability of indicated cell lines measured by CCK-8 assay after 24-hour treatment with elesclomol (ES). **F** Viability of indicated cell lines measured by CCK-8 assay after 24-h treatment with disulfiram (DSF) (plus equimolar CuCl₂). **G** Viability of indicated cells measured by CCK-8 assay after 24-h treatment with ES (with or without 10 μM tetrathiomolybdate (TTM)). **H** Viability of indicated cells measured by CCK-8 assay after 24-hour treatment with DSF (with or without 10 μM TTM). Data are presented as mean ± standard deviation (SD). Three independent experiments were performed. *P < 0.05, **P < 0.01, ***P < 0.001. (TCGA The Cancer Genome Atlas, THCA Thyroid Carcinoma).

Disturbances of copper homeostasis have emerged as a hallmark of thyroid carcinoma [7, 23, 24]. Analysis of both unmatched and matched thyroid cancer datasets from The Cancer Genome Atlas (TCGA) revealed aberrant regulation of key molecular pathways governing copper homeostasis (Fig. 1A, C), as evidenced by altered expression patterns of genes controlling copper acquisition, transport, storage, and efflux [23]. Notably, substantial downregulation of metallothioneins proteins [25], critical for copper storage, was observed in tumor tissues (Fig. 1B, D), indicating compromised copper buffering capacity in thyroid cancer cells. These findings suggest a latent vulnerability to copper-induced stress due to dysregulated copper metabolism. This vulnerability is further supported by the significantly enhanced sensitivity of thyroid cancer cells to copper ionophores elesclomol (ES) and disulfiram (DSF) compared to normal thyroid cells (Fig. 1E, F). Critically, the inhibitory effects of ES and DSF were readily reversed by the copper chelator tetrathiomolybdate (TTM) (Fig. 1G, H), confirming cuproptosis as the fundamental mechanism underlying their cytotoxicity. Collectively, these results highlight the therapeutic potential of targeting copper homeostasis with copper ionophores for thyroid cancer treatment.

### Sunitinib enhances cuproptosis induced by copper ionophores in thyroid cancer

**Fig. 2:**
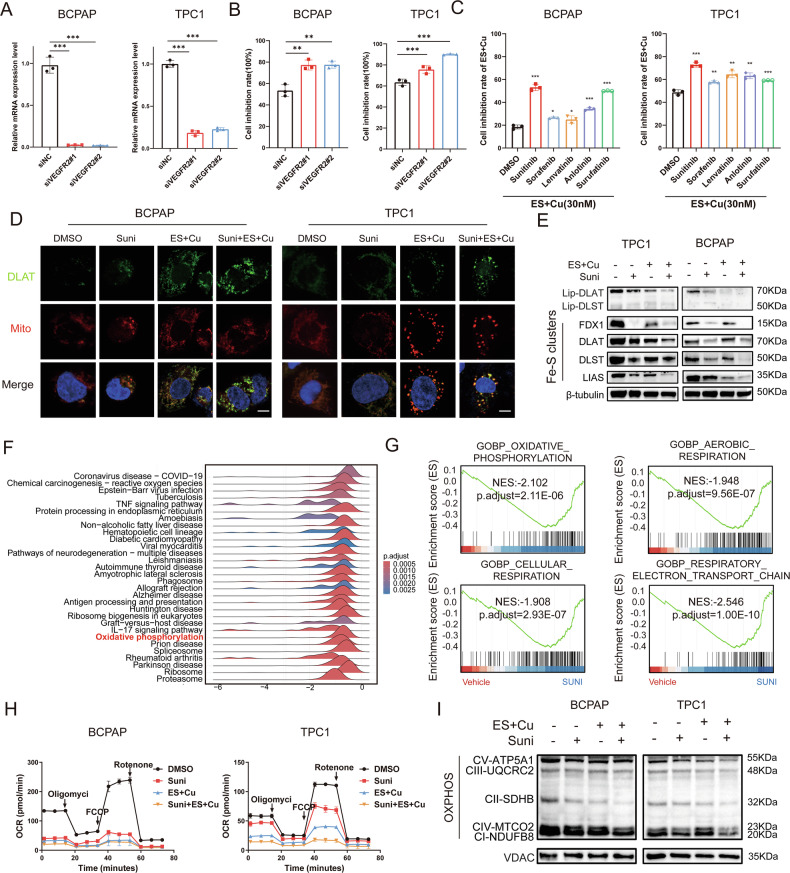
Sunitinib enhances sensitivity to ES&Cu-Induced cuproptosis in PTC Cells. **A** QPCR quantification confirmed efficient VEGFR2 knockdown (*n* = 3 independent experiments; two-tailed Student’s t-test). **B** Relative sensitivity to ES&Cu following VEGFR2 silencing (ES&Cu at 30 nM). **C** Sensitivity of B-CPAP and TPC-1 cells to ES&Cu in combination with five VEGFR2-targeted tyrosine kinase inhibitors (TKIs) (ES&Cu at 30 nM). **D** Immunofluorescence (IF) imaging of DLAT aggregation (green) in B-CPAP and TPC-1cells treated for 24 h with:DMSO (control), 5 μM Sunitinib (Suni), 50 nM ES&Cu, Suni + ES&Cu (red, Mitotracker; blue, DAPI, Scale bars: 5 μm). **E** Western blot analysis of Fe-S cluster proteins (FDX1, LIAS, DLAT, DLST) and DLAT lipoylation in cells treated with Suni, ES&Cu, or their combination. **F** Gene Set Enrichment Analysis (GSEA) of RNA-seq data revealed significant downregulation of the oxidative phosphorylation (OXPHOS) pathway in Sunitinib-treated cells, as visualized by the characteristic mountain plot. **G** GSEA of gene sets associated with oxidative phosphorylation, aerobic respiration, cellular respiration, and respiration electron transport chain between Sunitinib-treated versus vehicle-treated cells. **H** Seahorse extracellular flux analysis confirming further impairment of mitochondrial OXPHOS function with Sunitinib/ES&Cu combination therapy. **I** Western blot analysis of electron transport chain complex activity following combination treatment. Data are presented as mean ± standard deviation (SD). Three independent experiments were performed. *P < 0.05, **P < 0.01, ***P < 0.001.

Based on established crosstalk between copper metabolism and VEGF signaling, we experimentally evaluated whether VEGFR2 silencing modulates cuproptosis sensitivity in thyroid carcinoma cells. Consistent with our hypothesis, RNA interference targeting VEGFR2 significantly upregulated sensitivity to cuproptosis inducer elesclomol plus Cu²⁺ (ES&Cu) in B-CPAP and TPC-1 cells (Fig. 2A–B). Therefore, we propose to investigate the therapeutic potential of combining VEGFR2 inhibitors with cuproptosis inducers in thyroid carcinoma. As VEGFR2 monoclonal antibodies remain clinically underexplored in thyroid cancer, we screened five multi-target VEGFR2 tyrosine kinase inhibitors (TKIs) used in thyroid oncology for synergy with ES&Cu. Each TKI was used at the concentration of IC50 (Supplementary Fig. S1A). Aligning with our initial postulate, most VEGFR2-targeting TKIs demonstrated cuproptosis-promoting effects, among which Sunitinib exhibiting the most pronounced synergistic effect in cuproptosis potentiation, was selected for subsequent mechanistic exploration (Fig. 2C). Sunitinib, an oral multi-target TKI, has garnered attention in thyroid cancer therapy, particularly for iodine-refractory well-differentiated thyroid carcinoma (WDTC) and medullary thyroid carcinoma (MTC) [26, 27]. Long-term clonogenic assay showed synergy between Sunitinib and ES+Cu (Supplementary Fig. S1B). Moreover, Sunitinib combined with ES&Cu induced sustained suppression of papillary thyroid carcinoma (PTC) cell proliferation (Supplementary Fig. S1C). Synergy quantification via combination index (CI) analysis showed moderate-to-strong synergism (CI < 1.0) in B-CPAP and TPC-1 cell lines (Supplementary Fig. S1D). Notably, Sunitinib consistently sensitized diverse pathological subtypes of thyroid cancer cells to cuproptosis (Supplementary Fig. S1E).

To elucidate the mechanism underlying Sunitinib-mediated sensitization to ES&Cu, we employed pharmacological inhibitors targeting distinct cell death pathways: ferrostatin-1 (ferroptosis), necrostatin-1 (necroptosis), Z-VAD-FMK (apoptosis), and tetrathiomolybdate (TTM, copper chelation). Co-treatment with Sunitinib and ES&Cu significantly amplified both apoptosis and cuproptosis compared to ES&Cu monotherapy (Supplementary Fig. S2A). Flow cytometry confirmed an increase in apoptotic cell populations with combination therapy (Supplementary Fig. S2B).

Given that Sunitinib may sensitiz ES+Cu through multiple mechanisms and apoptosis was a well-established form of cell death induced by Sunitinib, we are particularly interested in elucidating the mechanisms of enhanced cuproptosis. As a newly defined regulated cell death modality, cuproptosis is characterized by aggregation of lipoylated proteins such as dihydrolipoamide S-acetyltransferase (DLAT) [28]. Immunofluorescence (IF) imaging revealed enhanced DLAT oligomerization in Sunitinib/ES&Cu-treated cells versus ES&Cu alone (Fig. 2D). Co-localization analysis and Semi-quantitative outcomes of IF imaging of DLAT aggregation (green) were shown in Supplementary Fig. S3A–B. Concurrently, reduced lipoylation of DLAT and DLST, a hallmark of cuproptosis—were observed in combination-treated cells (Fig. 2E, Supplementary Fig. S3C). Downregulated mitochondrial Fe-S cluster proteins (including FDX1, LIAS, DLAT and DLST), another hallmark of cuproptosis, was also observed (Fig. 2E). To further explore the effect of Sunitinib on mitochondrial function, we performed RNA sequencing analysis on B-CPAP cells treated with or without Sunitinib, and GSEA analysis showed reduced oxidative phosphorylation (OXPHOS) levels in cells treated with Sunitinib (Fig. 2F). Further analysis revealed a robust association between Sunitinib treatment and gene sets implicated in OXPHOS, mitochondrial aerobic respiration, and the respiratory electron transport chain (Fig. 2G). These findings collectively suggest a potential role for metabolic reprogramming in Sunitinib response. Seahorse experiments confirmed that the combination of Sunitinib further reduced mitochondrial oxidative phosphorylation function (Fig. 2H, Supplementary Fig. S4A). Past literature has shown that copper death reduces mitochondrial function, including reduced expression of mitochondrial respiratory chain complexes and intracellular ATP content [29]. We detected the expression of mitochondrial respiratory chain complexes with Western blot after combination Sunitinib with ES&Cu (Fig. 2I, Supplementary Fig. S4B) and mitochondrial complex II was found to be the most significantly decreased after combination with Sunitinib. Heat map showed changes in mRNA levels of the lipoic acid pathway, PDH complex, and Fe-S cluster genes (Supplementary Fig. S4C). The combination of Sunitinib with ES&Cu further reduced intracellular ATP content (Supplementary Fig. S4D). These findings validate the enhancement of cuproptosis in thyroid cancer by Sunitinib plus ES&Cu treatment.

### Sunitinib enhances ES&Cu-induced cuproptosis via SLC31A1 upregulation

**Fig. 3:**
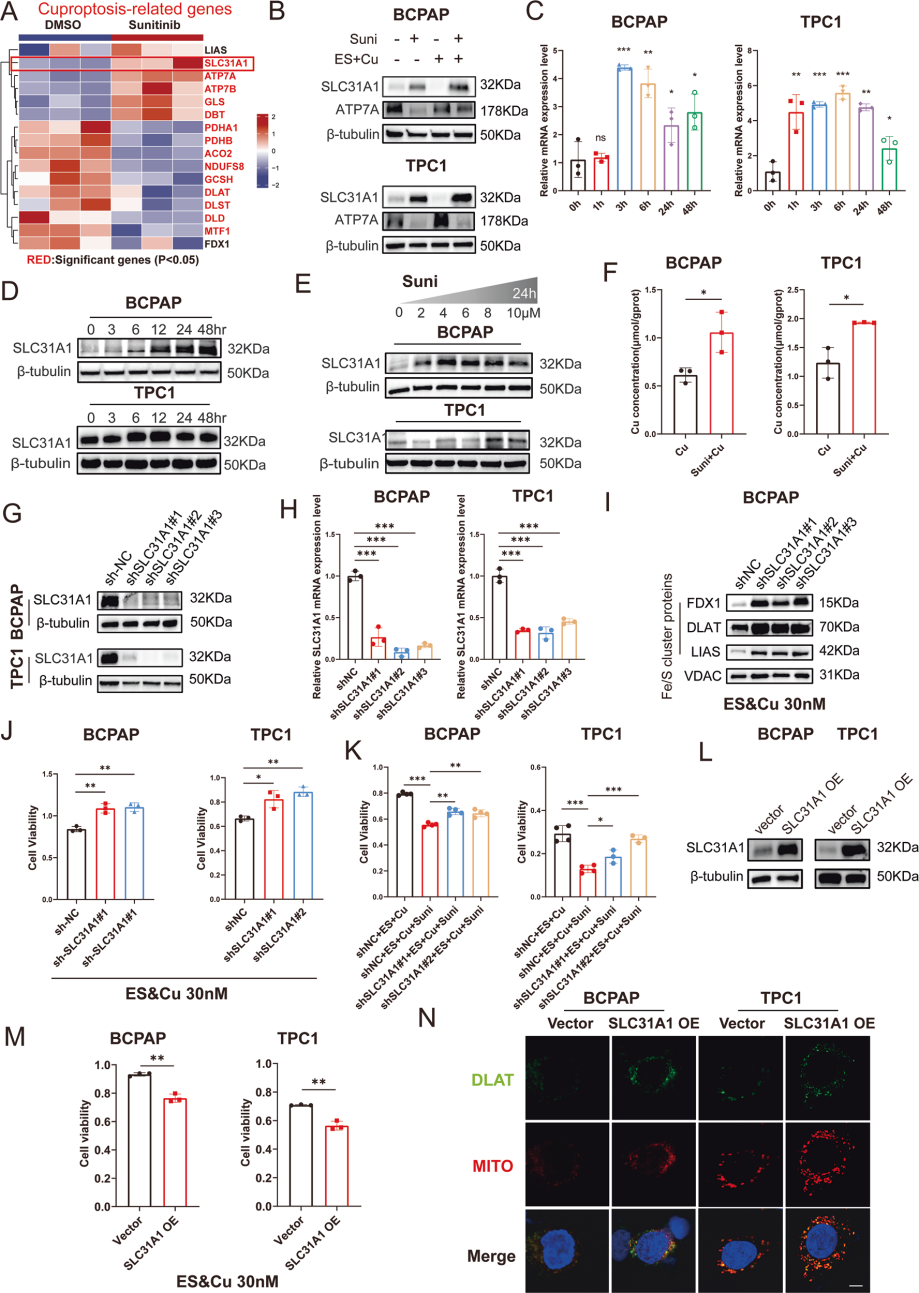
Sunitinib sensitizes PTC cells to ES&Cu-induced cuproptosis via SLC31A1 upregulation. **A** Heatmap of RNA sequencing data showing expression changes of cuproptosis-related genes following Sunitinib treatment. **B** Protein levels of SLC31A1/ATP7A assessed by Western blot (WB) after treatment with Sunitinib, ES&Cu, or their combination. Time-dependent increases in SLC31A1 mRNA (**C**) and protein (**D**) levels following Sunitinib exposure. **E** Concentration-dependent increases in SLC31A1 protein levels following 24 h Sunitinib exposure. **F** Elevated intracellular copper content in Sunitinib-treated cells. Validation of SLC31A1 knockdown efficiency via shRNA in B-CPAP and TPC-1 cells, confirmed by WB (**G**) and qPCR (**H**). **I** Increased Fe-S cluster protein levels (FDX1, DLAT, LIAS) in SLC31A1-knockdowned cells. **J** CCK-8 assay demonstrating enhanced resistance to ES&Cu-induced cuproptosis after SLC31A1 knockdown in B-CPAP and TPC-1 cells. **K** Rescue experiments demonstrating partial reversal of cuproptosis sensitivity in SLC31A1- knockdowned cells. **L** Validation of SLC31A1 overexpression in B-CPAP and TPC-1 cells, confirmed by WB. **M** CCK-8 assay demonstrating enhanced sensitivity to ES&Cu-induced cuproptosis after SLC31A1 overexpression in B-CPAP and TPC-1 cells. **N** Immunofluorescence quantification revealing increased DLAT puncta formation after SLC31A1 overexpression. Scale bar: 5 μm. Data are presented as mean ± standard deviation (SD). Three independent experiments were performed. *P < 0.05, **P < 0.01, ***P < 0.001.

Heatmap analysis of cuproptosis-related genes revealed increased SLC31A1 expression in Sunitinib-treated thyroid cancer cells (Fig. 3A). Western blot (WB) confirmed elevated SLC31A1 protein following Sunitinib treatment, align with result of RNA-seq analysis (Fig. 3B), with protein and mRNA expression proportional to treatment duration (Fig. 3C–D). The protein level of SLC31A1 was also gradually elevated in response to increasing concentrations of Sunitinib (Fig. 3E). Consistent with SLC31A1’s role in copper uptake, intracellular copper content increased after Sunitinib exposure (Fig. 3F). Stable SLC31A1 knockdown using shRNA in B-CPAP and TPC-1 cells (Fig. 3G–H) reduced ES&Cu-induced cuproptosis, as evidenced by elevated Fe-S cluster protein levels (Fig. 3I, Supplementary Fig. S5A) and resistance in viability assays (Fig. 3J). Depletion of SLC31A1 partially abolished the sensitizing effect of Sunitinib on cuproptosis (Fig. 3K). We also repeated the rescue experiments with cell death inhibitors under SLC31A1 knockdown conditions and found that upon SLC31A1 knockdown (Supplementary Fig. S5B), the rescue effect of the cuproptosis inhibitor TTM was markedly diminished, while no significant rescue difference was observed with other cell death inhibitors. These results suggest that SLC31A1 primarily contributes to the Sunitinib-induced sensitization of cuproptosis. To further investigate the functional role of SLC31A1, we established stable overexpression cell lines. As validated by Western blot (Fig. 3L), SLC31A1 overexpression sensitized both BCPAP and TPC1 cells to the cuproptosis inducer ES&Cu, as evidenced by reduced cell viability upon adminstration of ES&Cu (30nM) (Fig. 3M). Furthermore, under treatment with ES+Cu (30 nM), SLC31A1 overexpression enhanced the oligomerization of DLAT (Fig. 3N). Co-localization analysis and Semi-quantitative outcomes of IF imaging of DLAT aggregation (green) were shown in Supplementary Fig. S5C–D.

To evaluate the clinical relevance of SLC31A1 in thyroid carcinoma, we initially analyzed its expression patterns and associations with clinicopathological features in TCGA data. Pan-cancer analysis revealed differential SLC31A1 expression across cancer types (Supplementary Fig. S6A). In thyroid carcinoma, SLC31A1 levels were significantly downregulated in tumor tissues compared to adjacent normal tissues (Supplementary Fig. S6B), with further reductions observed in advanced-stage tumors and those with lymph node metastasis (Supplementary Figs. S6C–D). Transwell assays confirmed that SLC31A1 knockdown enhanced the invasive capacity of B-CPAP and TPC-1 cells (Supplementary Fig. S6E), underscoring its role in suppressing tumor aggressiveness. Further analysis demonstrated that low SLC31A1 expression correlated with worse T stage, N stage, extrathyroidal extension, and aggressive histological subtypes (Table 1). Given that Sunitinib upregulates SLC31A1, the combination of Sunitinib and ES&Cu may offer therapeutic benefits for advanced and metastatic thyroid cancers. These findings position Sunitinib/ES&Cu combination therapy as a promising strategy to counteract SLC31A1 deficiency in aggressive thyroid carcinomas, leveraging Sunitinib’s capacity to restore copper uptake and amplify cuproptosis, although this hypothesis requires validation in future clinical trials.

**Table 1:** The correlation between the level of SLC31A1 expression and clinical characteristics of thyroid cancer in TCGA database.

Characteristics	SLC31A1-Low (*N* = 256)	SLC31A1-High (*N* = 256)	*P*value
Gender, *n* (%)			
Female	185 (36.1%)	188 (36.7%)	0.766
Male	71 (13.9%)	68 (13.3%)	
Age, *n* (%)			
<= 45	116 (22.7%)	127 (24.8%)	0.330
> 45	140 (27.3%)	129 (25.2%)	
Pathologic T stage, *n* (%)			
T1&T2	139 (27.3%)	173 (33.9%)	**0.002**
T3&T4	116 (22.7%)	82 (16.1%)	
Pathologic *N* stage, *n* (%)			
N0	103 (22.3%)	126 (27.3%)	**0.004**
N1	136 (29.4%)	97 (21%)	
Pathologic *M* stage, *n* (%)			
M0	149 (50.5%)	137 (46.4%)	1.000
M1	5 (1.7%)	4 (1.4%)	
Pathologic stage, *n* (%)			
Stage I&Stage II	160 (31.4%)	180 (35.3%)	0.060
Stage III&Stage IV	95 (18.6%)	75 (14.7%)	
Neoplasm location, *n* (%)			
Left lobe&Right lobe	205 (40.5%)	191 (37.7%)	0.183
Isthmus	11 (2.2%)	11 (2.2%)	
Bilateral	36 (7.1%)	52 (10.3%)	
Extrathyroidal extension, *n* (%)			
No	155 (31.4%)	185 (37.4%)	**0.004**
Yes	92 (18.6%)	62 (12.6%)	
Histological type, *n* (%)			
Classical&Follicular	224 (43.8%)	243 (47.5%)	**0.003**
Other&Tall Cell	32 (6.2%)	13 (2.5%)	

Missing values were not included in the analysis. Bold text indicates a Pvalue less than 0.05.

### Sunitinib activates ATF3, a transcription factor of SLC31A1

**Fig. 4:**
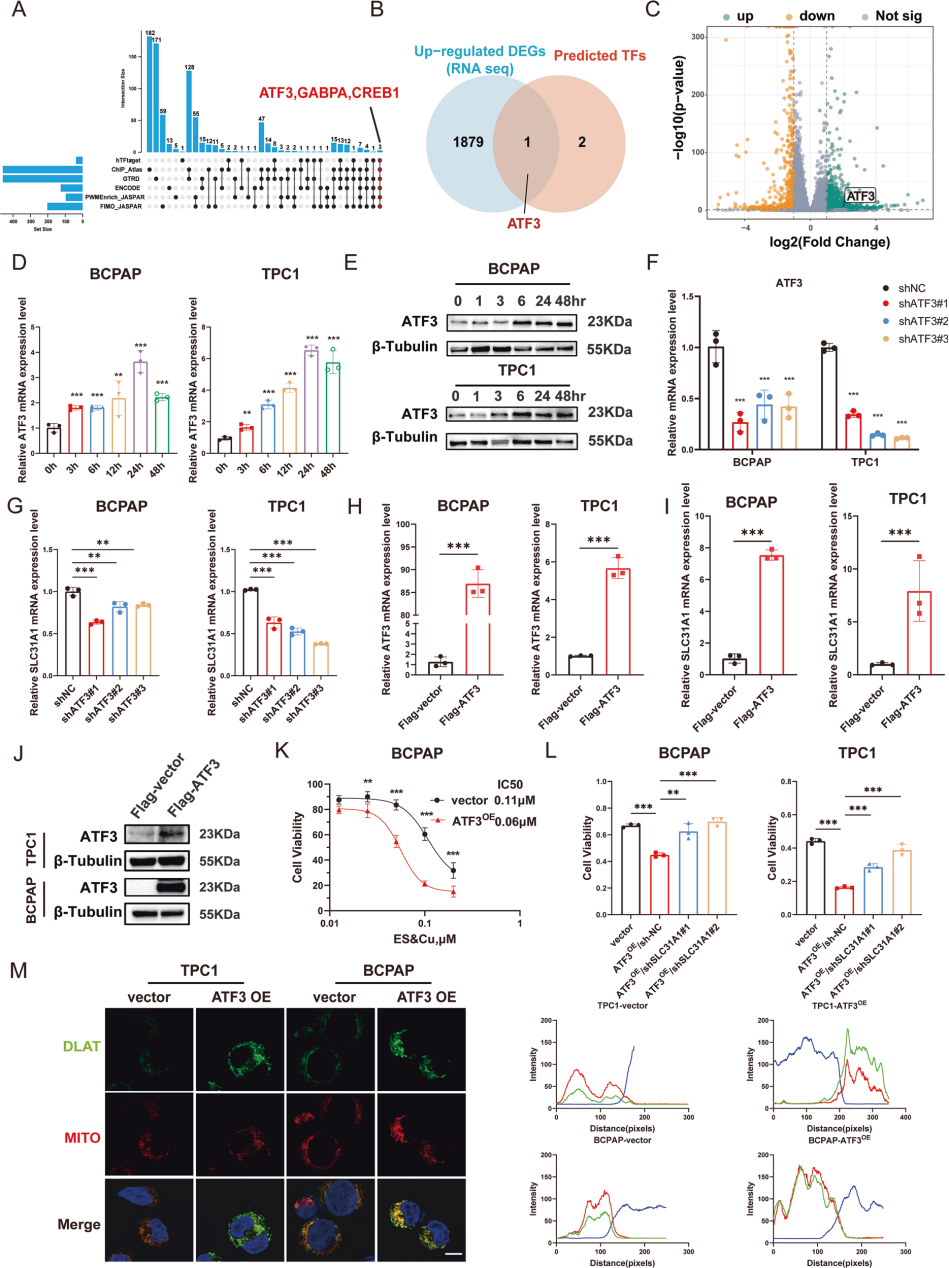
ATF3 mediates SLC31A1 upregulation to enhance cuproptosis sensitivity. **A** Upset plot illustrating candidate transcription factors (TFs) of SLC31A1 predicted by multi-database analysis (ENCODE, JASPAR, GTRD, etc.). **B** Venn diagram showing overlap between differentially expressed genes (DEGs) from RNA sequencing and predicted TFs, identifying ATF3 as the shared candidate. **C** Volcano plot of RNA-seq data demonstrating significant upregulation of ATF3 mRNA in Sunitinib-treated B-CPAP cells. Time-dependent increases in ATF3 mRNA (**D**) and protein (**E**) levels following Sunitinib treatment, validated by Western blot and qPCR. **F** Validation of ATF3 knockdown efficiency via shRNA in B-CPAP and TPC-1cells. **G** Reduced SLC31A1 mRNA levels in ATF3-knockdowned cells. **H** Confirmation of stable ATF3 mRNA overexpression in B-CPAP and TPC-1cells. **I** Increased SLC31A1 mRNA levels in ATF3-overexpressed cells. **J** Confirmation of stable ATF3 protein overexpression in B-CPAP and TPC-1cells. **K** IC50 shift quantification. ES&Cu IC50 decreased from 0.11 μM to 0.06 μM with ATF3 overexpression (*n* = 3; dose-response curve, four-parameter logistic fit). **L** Partial rescue of ATF3-mediated cuproptosis sensitization upon SLC31A1 knockdown, confirming the ATF3-SLC31A1 regulatory axis. **M** Immunofluorescence quantification revealing increased DLAT puncta formation after ATF3 overexpression. Scale bar: 5 μm. Data are presented as mean ± standard deviation (SD). Three independent experiments were performed. **P < 0.01, ***P < 0.001.

To elucidate the mechanism underlying Sunitinib-induced SLC31A1 upregulation in thyroid cancer, we hypothesized transcriptional activation of SLC31A1 based on its elevated mRNA and protein levels post-treatment. Integration of ENCODE, ChIP Atlas, JASPAR (via PWMEnrich and FIMO), GTRD, and hTFtarget databases identified three potential transcription factors (ATF3, GABPA, CREB1) regulating SLC31A1 (Fig. 4A). RNA-seq analysis of Sunitinib-treated cells revealed 1,880 upregulated genes, with ATF3 emerging as the sole transcription factor overlapping between predicted regulators and differentially expressed genes (Fig. 4B). Activating Transcription Factor 3 (ATF3), a constitutively expressed member of the ATF/CREB superfamily, serves as an immediate-early response transcription factor that orchestrates adaptive cellular responses to oxidative stress, genotoxic injury, and metabolic perturbation [30]. While ATF3 is known to regulate iron metabolism through SLC7A11 in ferroptosis [31, 32], its potential role in copper trafficking has yet to be elucidated.

Volcano plot analysis confirmed significant ATF3 upregulation following Sunitinib treatment (Fig. 4C). Analysis of paired and unpaired tumor/normal samples in the TCGA database revealed reduced ATF3 expression in thyroid carcinoma (Supplementary Fig. S7A–B). Time-dependent increases in ATF3 mRNA and protein levels were validated by qPCR and Western blot (Fig. 4D–E, Supplementary Fig. S7C). Stable knockdown of ATF3 via shRNA in B-CPAP and TPC-1 cells (Fig. 4F; Supplementary Fig. S7D) resulted in decreased SLC31A1 mRNA expression (Fig. 4G). Furthermore, upon ES&Cu treatment, these cells exhibited elevated Fe-S protein levels (Supplementary Fig. S7E). Conversely, ATF3 overexpression (Fig. 4H) enhanced SLC31A1 mRNA expression (Fig. 4I). The decrease in IC50 further suggests that ATF3 overexpression (Fig. 4J, Supplementary Fig. S7F) increases the sensitivity of B-CPAP and TPC-1 cells to cupropotosis (Fig. 4K, Supplementary Fig. S7G). Rescue experiments demonstrated partial reversal of ATF3-mediated cuproptosis sensitization upon SLC31A1 knockdown (Fig. 4L), while immunofluorescence imaging revealed enhanced DLAT oligomerization in ATF3-overexpression cells (Fig. 4M, Supplementary Fig. S7H), establishing a functional ATF3-SLC31A1 regulatory axis in Sunitinib’s mechanism. We also repeated the rescue experiments with cell death inhibitors under ATF3 knockdown conditions and found that upon ATF3 knockdown (Supplementary Fig. S7I), the rescue effect of the cuproptosis inhibitor TTM was markedly diminished, while no significant rescue difference was observed with other cell death inhibitors. These results suggest that ATF3 primarily contributes to the Sunitinib-induced sensitization of cuproptosis.

### ATF3 activates transcription by directly binding to the SLC31A1 Promoter

**Fig. 5:**
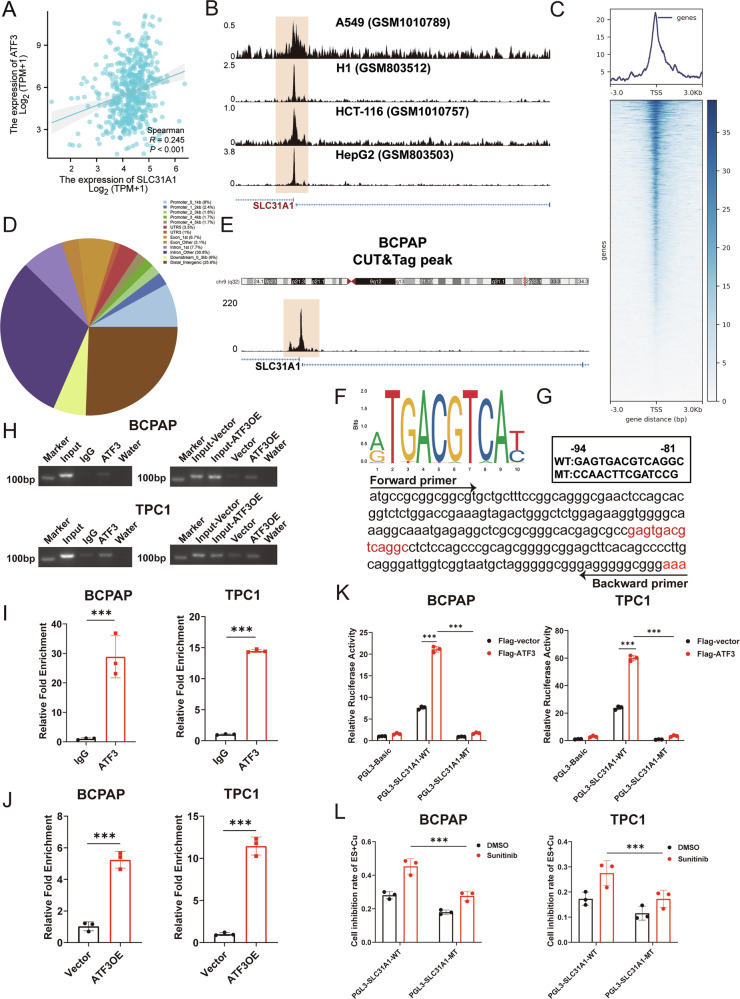
ATF3 activates transcription by directly binding to the SLC31A1 promoter. **A** Correlation analysis demonstrated a significant association between ATF3 and SLC31A1 in thyroid cancer. **B** Enrichment of ATF3 on the SLC31A1 promoter derived from CHIP-seq data in the GEO database. **C** Genome-wide CUT&Tag analysis in B-CPAP cells identified robust enrichment of ATF3 binding peaks. **D** Peak distribution analysis confirming promoter regions as primary binding sites. **E** Visualization of ATF3 binding at the SLC31A1 promoter via IGV. **F** JASPAR-predicted motifs in the proximal promoter region. **G** A putative ATF3 binding site (-94 to -81 relative to the transcription start site) was identified. **H**–**J** Chromatin immunoprecipitation (ChIP) assay performed with ATF3 antibody followed by detection of SLC31A1 promoter through gel electrophoresis and qPCR in B-CPAP and TPC-1cells. **K** Luciferase reporter assays using wild-type or mutated binding site plasmids confirmed that disruption of this motif abolished ATF3-mediated transcriptional activation in B-CPAP and TPC-1 cells, establishing direct ATF3 binding to the SLC31A1 promoter as the mechanistic basis for transcriptional regulation. **L** CCK-8 assay demonstrating sensitivity to ES&Cu-induced cuproptosis after Sunitinib exposure with transfection of PGL3-SLC31A1-WT or PGL3-SLC31A1-MT plasmids after 48 h in B-CPAP and TPC-1 cells. Data are presented as mean ± standard deviation (SD). Three independent experiments were performed. ***P < 0.001.

Correlation analysis of samples in the TCGA database demonstrated a significant association between ATF3 and SLC31A1 in thyroid cancer (Fig. 5A). Analysis of publicly available ChIP-seq datasets revealed significant enrichment of ATF3 binding within the promoter region of SLC31A1 across multiple cell lines, including A549, H1, HCT-116, and HepG2 (Fig. 5B). To validate transcriptional regulation, genome-wide CUT&Tag analysis was performed in B-CPAP cells. The binding density of ATF3 was visualized by deepTools: CUT&Tag peaks for ATF3 exhibited robust enrichment (Fig. 5C), with peak distribution analysis identifying gene promoter regions as primary binding sites (Fig. 5D). A distinct ATF3 binding peak was observed at the SLC31A1 promoter (Fig. 5E), aligning with JASPAR-predicted binding motifs in the proximal promoter region (Fig. 5F). A putative ATF3 binding site (-94 to -81 relative to the SLC31A1 transcription start site) was identified (Fig. 5G). Next, we performed chromatin immunoprecipitation (ChIP) assays in two PTC cell lines and found that the ATF3 antibody, rather than the IgG antibody, specifically pulled down the predicted SLC31A1 promoter fragment (Fig. 5H–I). In addition, ChIP assays with ATF3 overexpression cell lines exhibited higher binding to the SLC31A1 promoter than the control cells, and these findings were consistent with the results of the CUT&Tag assay in B-CPAP cells (Fig. 5H, J). Subsequently, a plasmid carrying sequences with mutated binding sites were designed, and luciferase reporter assays were conducted in B-CPAP and TPC-1 cells. Luciferase reporter assays using plasmids with wild-type or mutated binding sites confirmed that disruption of this motif abolished ATF3-mediated transcriptional activation in B-CPAP and TPC-1 cells (Fig. 5K), establishing direct ATF3 binding to the SLC31A1 promoter as the mechanistic basis for transcriptional activation. B-CPAP and TPC-1 cells were transiently transfected with either PGL3-SLC31A1-WT or PGL3-SLC31A1-MT plasmid constructs. Compared to cells transfected with the PGL3-SLC31A1-WT plasmid, those expressing the mutant construct exhibited significantly reduced sensitivity to ES-Cu co-treatment (*p* < 0.05). Furthermore, the sensitization of cells to ES&Cu by Sunitinib was significantly diminished in cells transfected with PGL3-SLC31A1-MT compared to wild-type controls. (Fig. 5L).

### Synergistic cytotoxic effects of copper ionophores and Sunitinib in xenograft mouse model

**Fig. 6:**
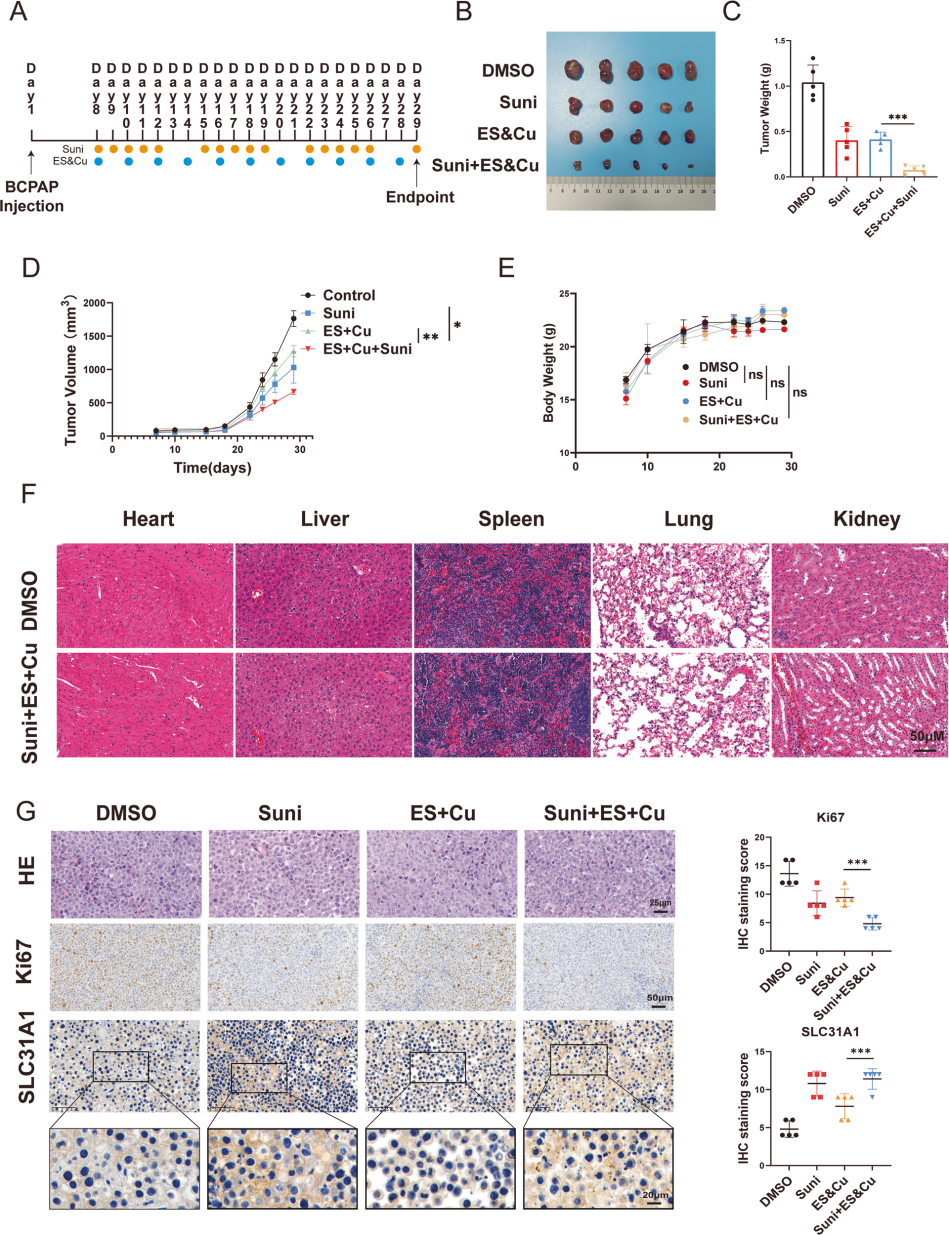
Sunitinib enhances ES&Cu-induced Cuproptosis Sensitivity in vivo. **A** Schematic of xenograft experimental design and drug administration protocol. **B**, **C** Combined Sunitinib and ES&Cu treatment markedly inhibits B-CPAP xenograft tumor growth, as shown by reduced average tumor volume (**B**) and weight (**C**). **D** Tumor growth kinetics. Longitudinal tumor volume measurements revealed synergistic inhibition: combination therapy significantly exceeding Sunitinib or ES&Cu alone. **E** Body weight monitoring. No significant treatment-related toxicity was observed, as evidenced by stable body weight trajectories. **F** H&E staining of major organs (heart, liver, spleen, lung, kidney) collected from mice after treatment. Scale bar = 50 μm. **G** Representative hematoxylin and eosin (H&E) staining, Ki-67 proliferation marker and SLC31A1 immunohistochemistry (IHC) in xenograft tumor sections. Data are presented as mean ± standard deviation (SD). Three independent experiments were performed. *P < 0.05, **P < 0.01, ***P < 0.001.

To translate these findings, we established thyroid cancer xenografts in 4-week-old nude mice subcutaneously inoculated with B-CPAP cells. Animals were randomized into four treatment cohorts: Vehicle control (DMSO, i.g., 5×/week); Sunitinib monotherapy (40 mg/kg, i.g., 5×/week); ES&Cu monotherapy (elesclomol 30 mg/kg + CuCl₂ 2 mg/kg, i.g., every other day.); Combination therapy (dosing as above) (Fig. 6A). After 20 days, combination treatment induced marked tumor regression, with significant reductions in tumor growth rate, volume and weight (Fig. 6B–D). No overt toxicity was observed, as evidenced by stable body weights across groups (Fig. 6E). No significant differences were observed in the histological morphology of major organs between the Suni+ES+Cu group and the control group, as assessed by hematoxylin and eosin (H&E) staining. These findings confirm the good biocompatibility of the treatment with normal tissues (Fig. 6F). Immunohistochemical analysis of Ki-67 revealed the lowest proliferation index in the combination cohort (Fig. 6G). Aligning with in vitro findings, immunohistochemistry of xenograft tumors showed increased SLC31A1 expression in Sunitinib-treated groups (Fig. 6G).

### Sunitinib upregulates ATF3 expression via ROS accumulation

**Fig. 7:**
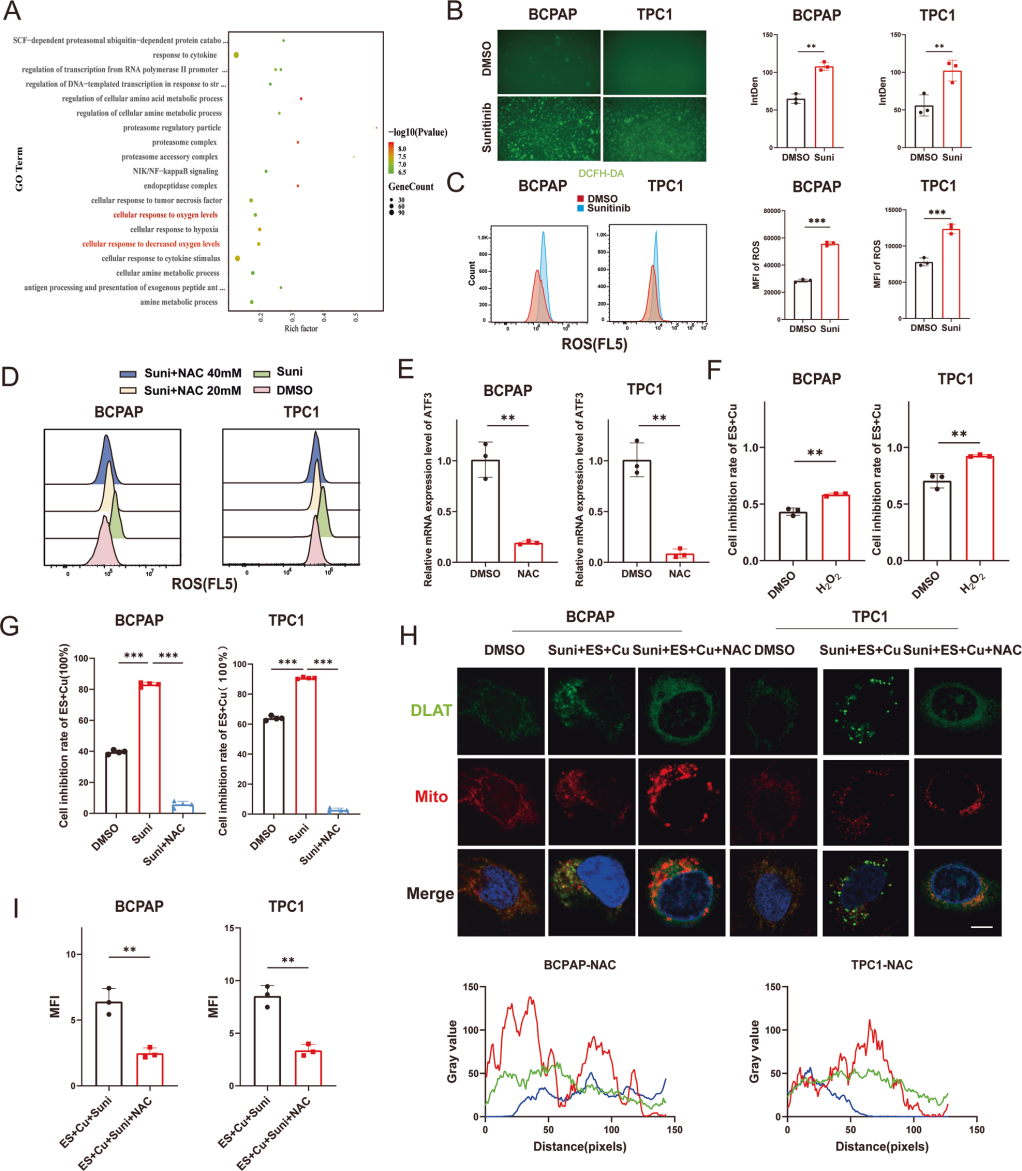
Sunitinib enhances cuproptosis sensitivity via ROS-dependent ATF3-SLC31A1 activation. **A** Cellular response to oxygen levels and cellular response to decreased oxygen levels pathways were enriched among the differential genes induced by Sunitinib through Gene Ontology (GO) analysis. **B**, **C** Intracellular ROS levels were elevated in Sunitinib-treated thyroid cancer cells. **D** Co-treatment with the ROS scavenger N-acetylcysteine (NAC) attenuated this ROS accumulation. **E** qPCR analysis demonstrated that NAC suppressed ATF3 mRNA levels in B-CPAP and TPC-1 cells. **F** Increased sensitivity to ES+Cu after exposure to H_2_O_2_ 24 h. **G** Rescue experiments revealed complete reversal of Sunitinib-enhanced cuproptosis sensitivity upon NAC administration. **H**. Immunofluorescence imaging showing reduced mitochondrial DLAT aggregation in NAC-Sunitinib-co-treated cells. Scale bar: 5 μm. Data are presented as mean ± standard deviation (SD). **I** Semi-quantitative outcomes derived from immunofluorescence analysis. Three independent experiments were performed. **P < 0.01, ***P < 0.001.

**Fig. 8:**
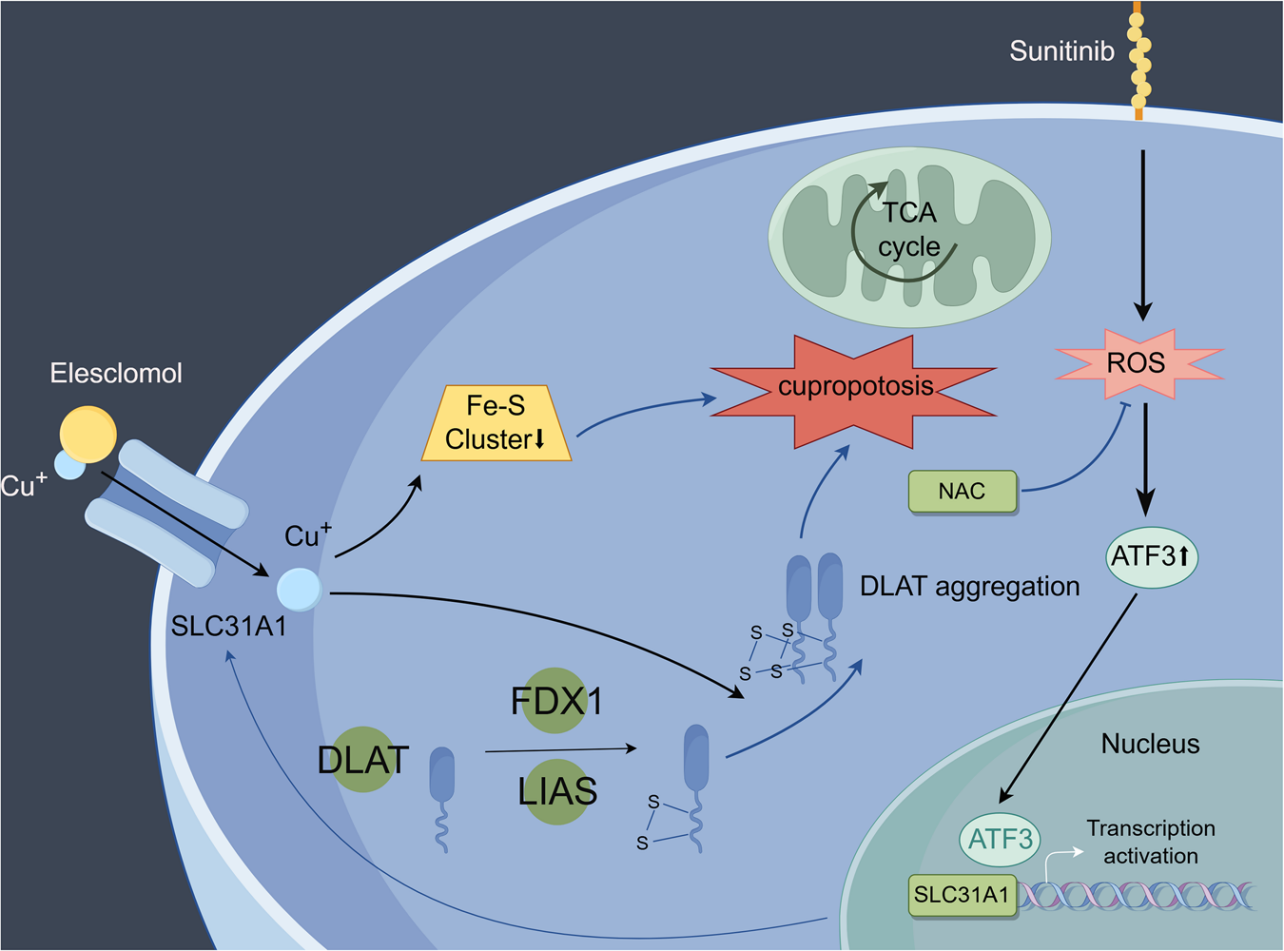
The schematic summarizes the proposed mechanism wherein Sunitinib-induced ROS accumulation activates the ATF3-SLC31A1 axis to amplify cuproptosis sensitivity in papillary thyroid carcinoma (by Figdraw).

ATF3 exhibits dichotomous, context-dependent functions in oncogenic processes, further complicated by its role as a transcriptional modulator of metallic homeostasis pathways [33]. Through Gene Ontology (GO) functional enrichment pathway analysis, we noticed the pathway of Cellular response to oxygen levels and cellular response to decreased oxygen levels pathways were enriched (*P* < 0.05) (Fig. 7A). Prior studies indicate that reactive oxygen species (ROS) activate ATF3 transcription via inducing DNA double strand breaks at transcription initiation region [34]. Therefore, we hypothesized that Sunitinib-induced ATF3 upregulation stems from ROS accumulation. Using DCFH-DA, we observed elevated intracellular ROS levels in Sunitinib-treated thyroid cancer cells (Fig. 7B–C). Flow cytometry confirmed that the ROS scavenger N-acetylcysteine (NAC) attenuated Sunitinib-driven ROS elevation (Fig. 7D). QPCR analysis revealed that NAC co-treatment suppressed ATF3 mRNA levels in B-CPAP cells (Fig. 7E), linking ROS to ATF3 regulation. To establish an oxidative stress model, cells were treated with 50 μM hydrogen peroxide (H₂O₂) for 24 h. This pre-conditioning resulted in a statistically significant increase in cellular sensitivity to elesclomol (ES) compared to untreated controls (*p* < 0.05) (Fig. 7F). Upon NAC administration, Sunitinib-enhanced cuproptosis sensitivity was completely reversed (Fig. 7G). Immunofluorescence further showed reduced DLAT aggregation in mitochondria after NAC co-treatment (Fig. 7H), corroborating ROS’s pivotal role in mediating cuproptosis potentiation. Semi-quantitative outcomes of IF imaging of DLAT aggregation (green) were shown in Fig. 7I. A schematic summarizes the Sunitinib-ROS-ATF3-SLC31A1 axis regulating cuproptosis sensitivity in papillary thyroid carcinoma (Fig. 8).

## Discussion

The development of TKIs combination regimens represents an evolving therapeutic paradigm for advanced and treatment-refractory thyroid carcinomas, seeking to augment antitumor efficacy and circumvent resistance mechanisms. Contemporary investigations highlight synergistic approaches integrating TKIs with complementary therapeutic modalities for surgically ineligible patients. One clinically validated strategy combines the multi-kinase inhibitor lenvatinib with the PD-1 immune checkpoint inhibitor pembrolizumab in radioiodine-refractory differentiated thyroid carcinoma (RAIR-DTC) [35]. This regimen demonstrated enhanced response sustainability versus lenvatinib monotherapy in progressive disease, establishing a second-line option for TKI-refractory cases. Concurrently, TKI co-administration with mutation-specific targeted agents is under clinical evaluation. Selective inhibition of somatic driver alterations alongside TKI therapy may extend progression-free survival, though such approaches currently lack regulatory approval for thyroid malignancies [36]. Additional investigative frameworks examine TKI combinations with cytotoxic chemotherapy or novel pathway-targeting compounds to overcome resistance mediated by genetic alterations and signaling pathway reactivation [2]. Collectively, these combinatorial strategies signify substantial progress in managing advanced thyroid cancer. Further validation through randomized controlled trials remains imperative to define their definitive role within the refractory thyroid cancer therapeutic algorithm [37].

Emerging evidence implicates copper homeostasis in VEGF signaling modulation, though molecular mechanisms and translational relevance remain incompletely characterized [38]. Cellular copper concentrations regulate tumor proliferation and proangiogenic enzyme activation, while the copper transporter SLC31A1 directly contributes to angiogenesis [39]. Mechanistically, endothelial SLC31A1 functions as a redox sensor independent of copper transport: VEGF-induced ROS promotes Cys189 sulfenylation, driving disulfide complex formation with VEGFR2 to propagate angiogenic signaling [21].

ScRNA-seq analyses further reveal SLC31A1’s involvement in immunomodulation and vascular remodeling pathways intersecting with VEGFR2 [40]. This copper-VEGF crosstalk influences tumor progression through angiogenesis, immune regulation, and metastatic processes. We propose targeted VEGFR2 inhibition perturbs copper homeostasis, demonstrating that Sunitinib synergistically potentiates copper ionophore-induced cuproptosis in thyroid carcinoma. This aligns with the past work by Cai Y et al. that identified a cuproptosis-related long non-coding RNA (lncRNA) prognostic model demonstrating significant correlations with sensitivity to targeted therapies, including 5-Fluorouracil, Bleomycin, Rapamycin, and notably, Sunitinib [41].

In this study, through integrated multi-omics analysis, rescue experiments, and clinical data mining, we systematically elucidate the molecular mechanism by which Sunitinib sensitizes cells to cuproptosis via the “ROS-ATF3-SLC31A1” axis. Furthermore, while SLC31A1 is established as a critical copper uptake transporter, its expression profile, prognostic relevance, and transcriptional regulatory network in thyroid carcinoma are poorly characterized. We further establish correlations between SLC31A1 expression levels and clinical parameters including tumor staging, metastatic status, and treatment sensitivity, thereby identifying novel therapeutic targets and precision oncology strategies for advanced thyroid carcinoma.

While our work provides mechanistic insights that kinase inhibitors can reprogram copper homeostasis via transcriptional regulation, expanding Sunitinib’s utility beyond angiogenesis inhibition, several limitations warrant consideration. First, the exclusive use of B-CPAP and TPC-1 cells may not fully recapitulate the biology of aggressive variants such as anaplastic thyroid cancer. Future validation in patient-derived organoids and orthotopic models of advanced subtypes is essential. Second, the long-term safety profile of copper accumulation, particularly systemic copper toxicity, remains uncharacterized beyond the 20-day treatment window. Extended Good Laboratory Practice (GLP)-compliant toxicology studies (e.g., 90-day trials) with rigorous serum copper monitoring are critical for clinical translation. Third, while Sunitinib-induced ROS accumulation was identified as a key driver of ATF3 activation, the precise molecular mechanisms underlying ROS upregulation require further elucidation. Addressing these gaps will strengthen the translational relevance of this therapeutic strategy. Notwithstanding these constraints, our study provides a preliminary investigation into the therapeutic potential of Sunitinib/Elesclomol combination therapy for thyroid carcinoma, holding promise for addressing unmet clinical needs in refractory disease settings.

## Conclusion

This study systematically elucidates the molecular mechanisms by which Sunitinib enhances ES&Cu-induced cuproptosis through multi-dimensional regulation. Specifically, we identify the “ROS-ATF3-SLC31A1” signaling axis as a key mediator that sensitizes thyroid cancer cells to cuproptosis. Taken together, these findings provide a novel conceptual framework for repurposing multi-target kinase inhibitors in thyroid cancer therapy.

## Supplementary information

SUPPLEMENTAL MATERIAL

WB original data-revised

## Data Availability

Datasets used and analyzed during the current study are available from the corresponding author upon reasonable request.

## Materials availability

Datasets used and analyzed during the current study are available from the corresponding author upon reasonable request.
