# Supplementary material for: Sunitinib enhances cuproptosis induced by copper ionophores via ROS-ATF3-SLC31A1 axis in thyroid Carcinoma
Source: Cell Death Dis. 2026 Jun 1;17(1):681. doi: 10.1038/s41419-026-08878-9 (PMC13434697; doi:10.1038/s41419-026-08878-9)

Fig2.E

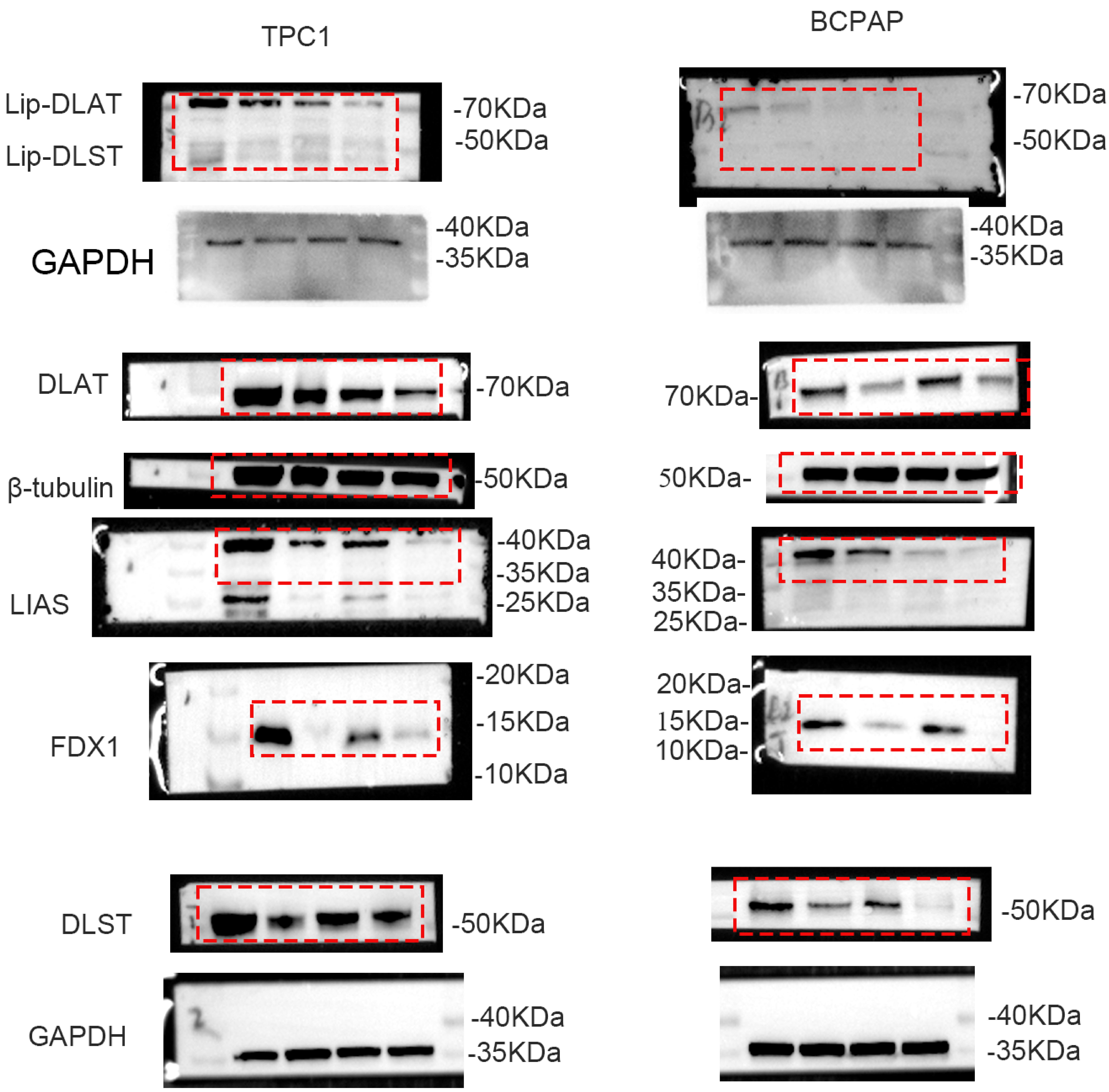
TPC1
BCPAP
Lip-DLAT
Lip-DLST
-70KDa
-50KDa
GAPDH
-40KDa
-35KDa
DLAT
70KDa-
β-tubulin
50KDa-
LIAS
-25KDa
FDX1
-20KDa
-15KDa
-10KDa
DLST
GAPDH

Fig2.I

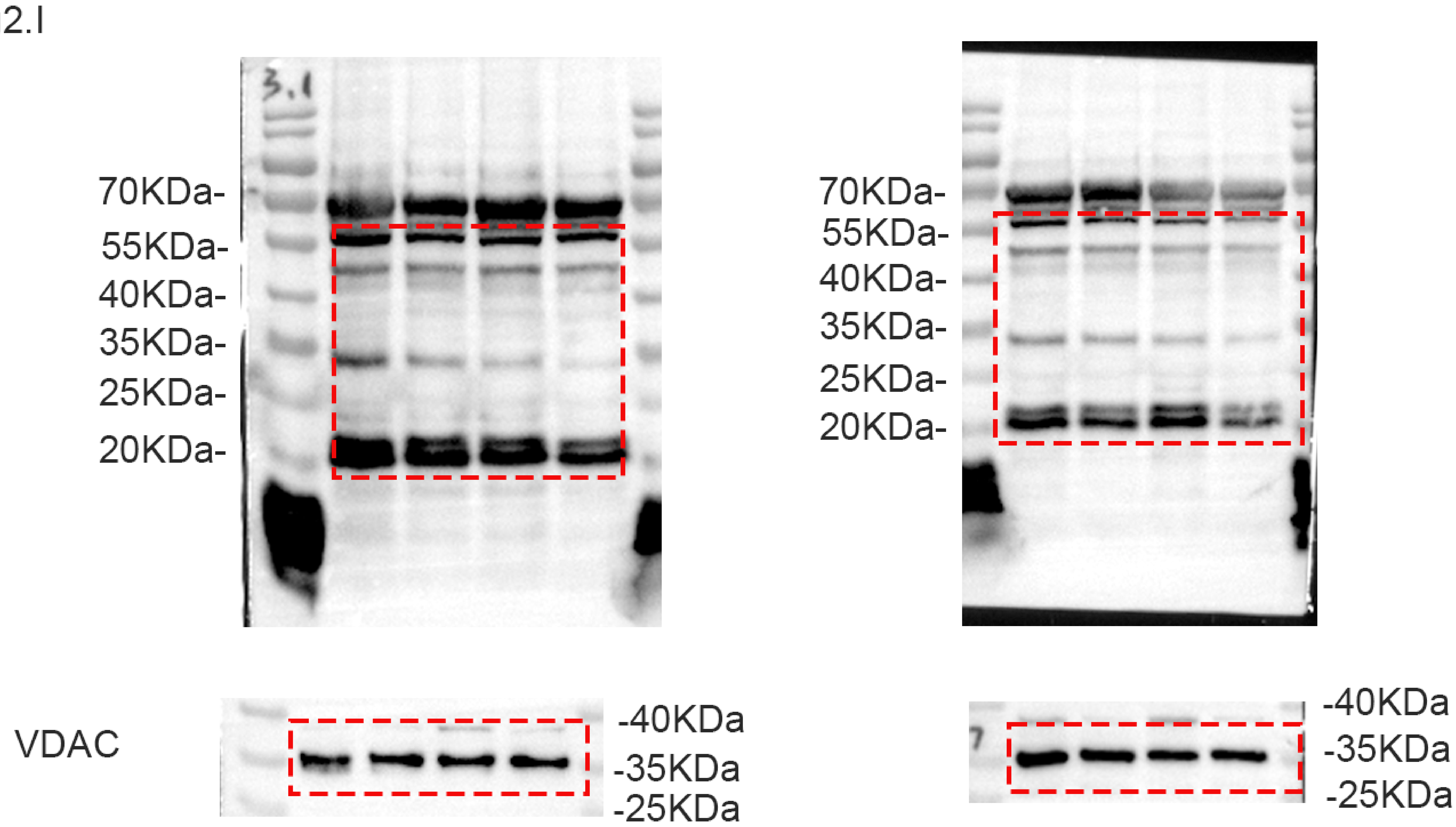
70KDa-
55KDa-
40KDa-
35KDa-
25KDa-
20KDa-
VDAC
-40KDa
-35KDa
-25KDa

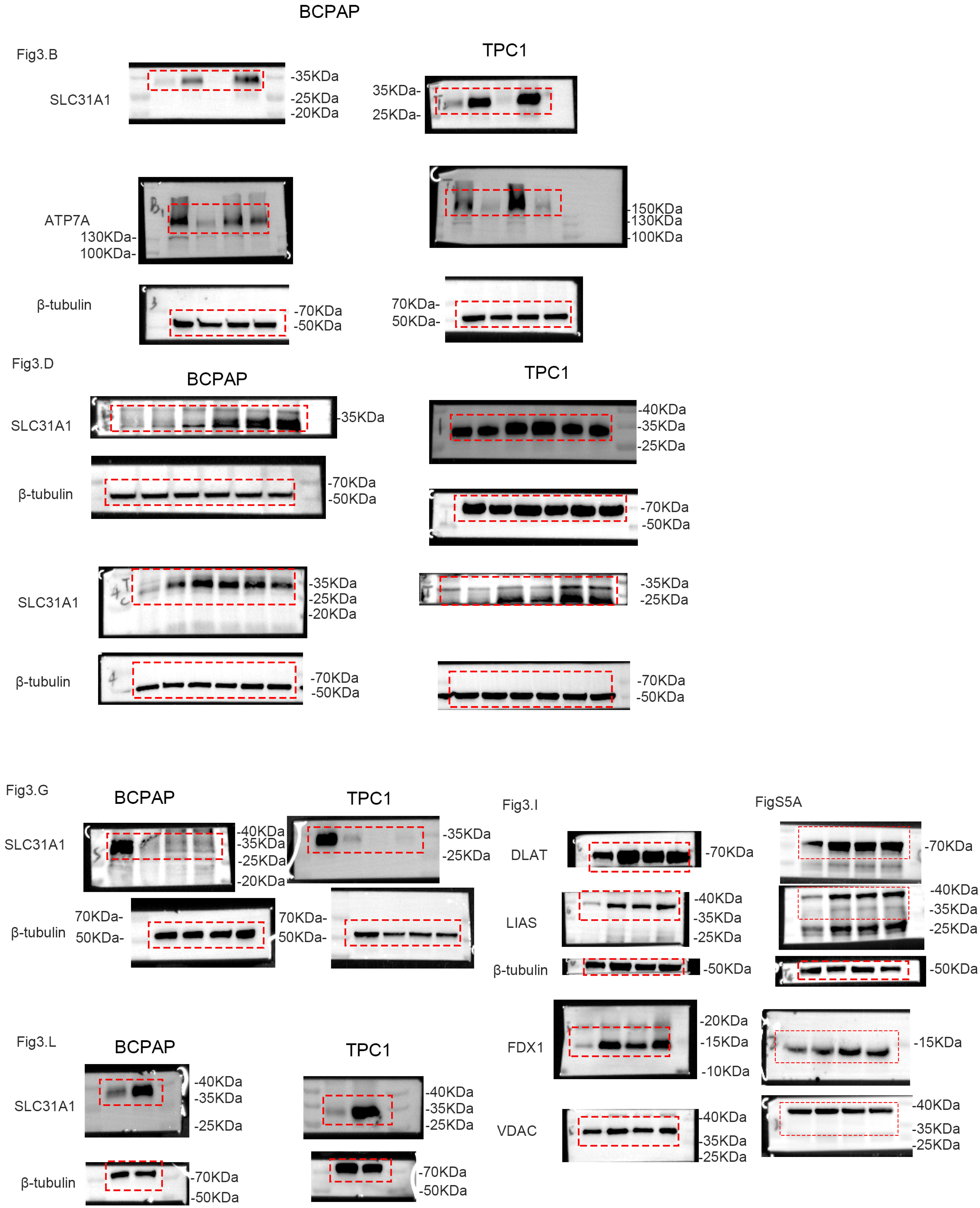
BCPAP
Fig3.B
TPC1
SLC31A1
-35KDa
-25KDa
-20KDa
35KDa-
25KDa-
ATP7A
130KDa-
100KDa-
-150KDa
-130KDa
-100KDa
β-tubulin
-70KDa
-50KDa
70KDa-
50KDa-
Fig3.D
BCPAP
TPC1
SLC31A1
-35KDa
-40KDa
-35KDa
-25KDa
β-tubulin
-70KDa
-50KDa
-70KDa
-50KDa
SLC31A1
-35KDa
-25KDa
-20KDa
-35KDa
-25KDa
β-tubulin
-70KDa
-50KDa
-70KDa
-50KDa
Fig3.G
BCPAP
TPC1
SLC31A1
-40KDa
-35KDa
-25KDa
-20KDa
-35KDa
-25KDa
β-tubulin
70KDa-
50KDa-
70KDa-
50KDa-
Fig3.I
FigS5A
DLAT
-70KDa
-70KDa
LIAS
-40KDa
-35KDa
-25KDa
-40KDa
-35KDa
-25KDa
β-tubulin
-50KDa
-50KDa
FDX1
-20KDa
-15KDa
-10KDa
-15KDa
VDAC
-40KDa
-35KDa
-25KDa
-40KDa
-35KDa
-25KDa
Fig3.L
BCPAP
TPC1
SLC31A1
-40KDa
-35KDa
-25KDa
-40KDa
-35KDa
-25KDa
β-tubulin
-70KDa
-50KDa
-70KDa
-50KDa

Fig4E

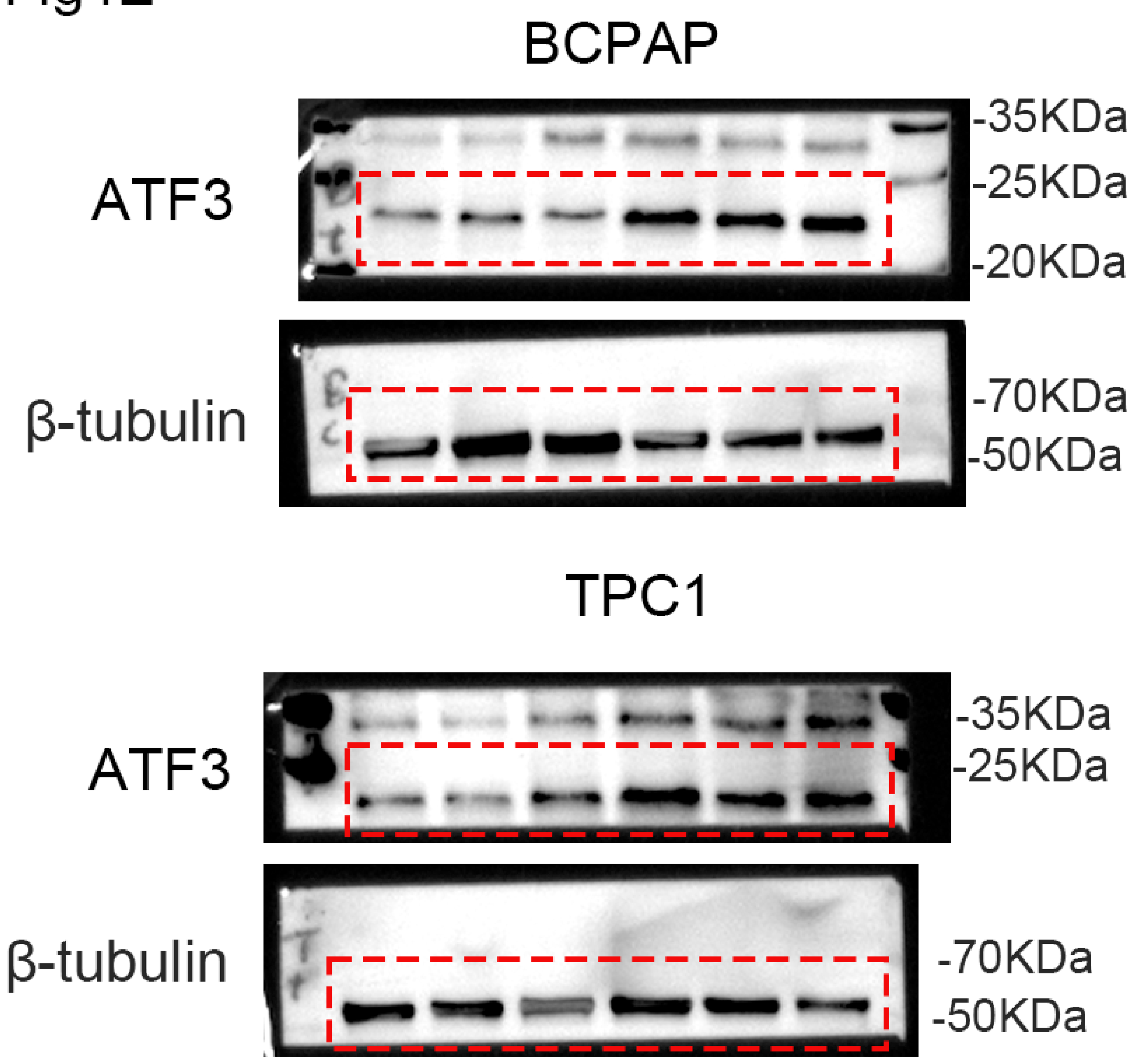

Fig4J

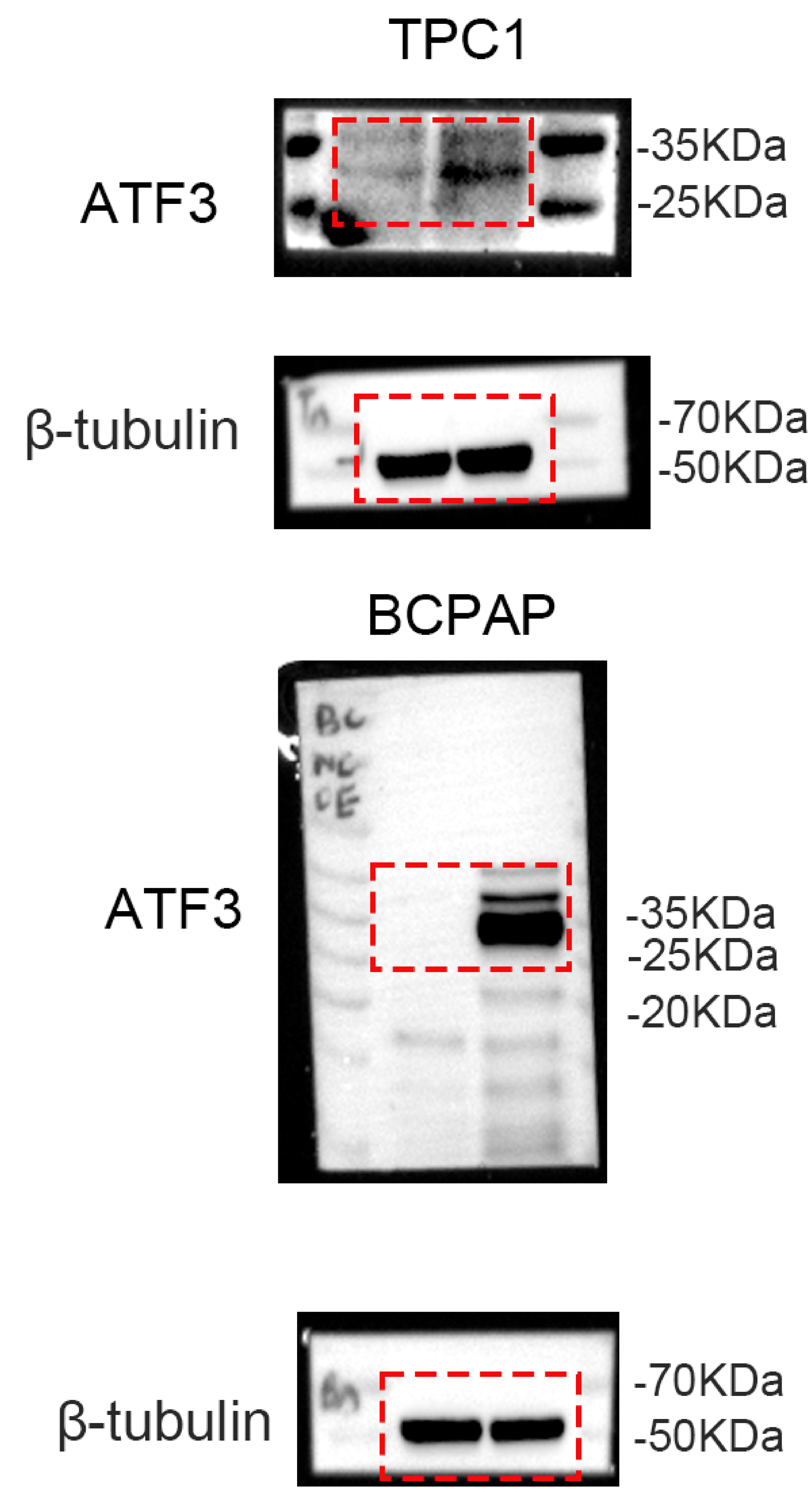

FigS6D

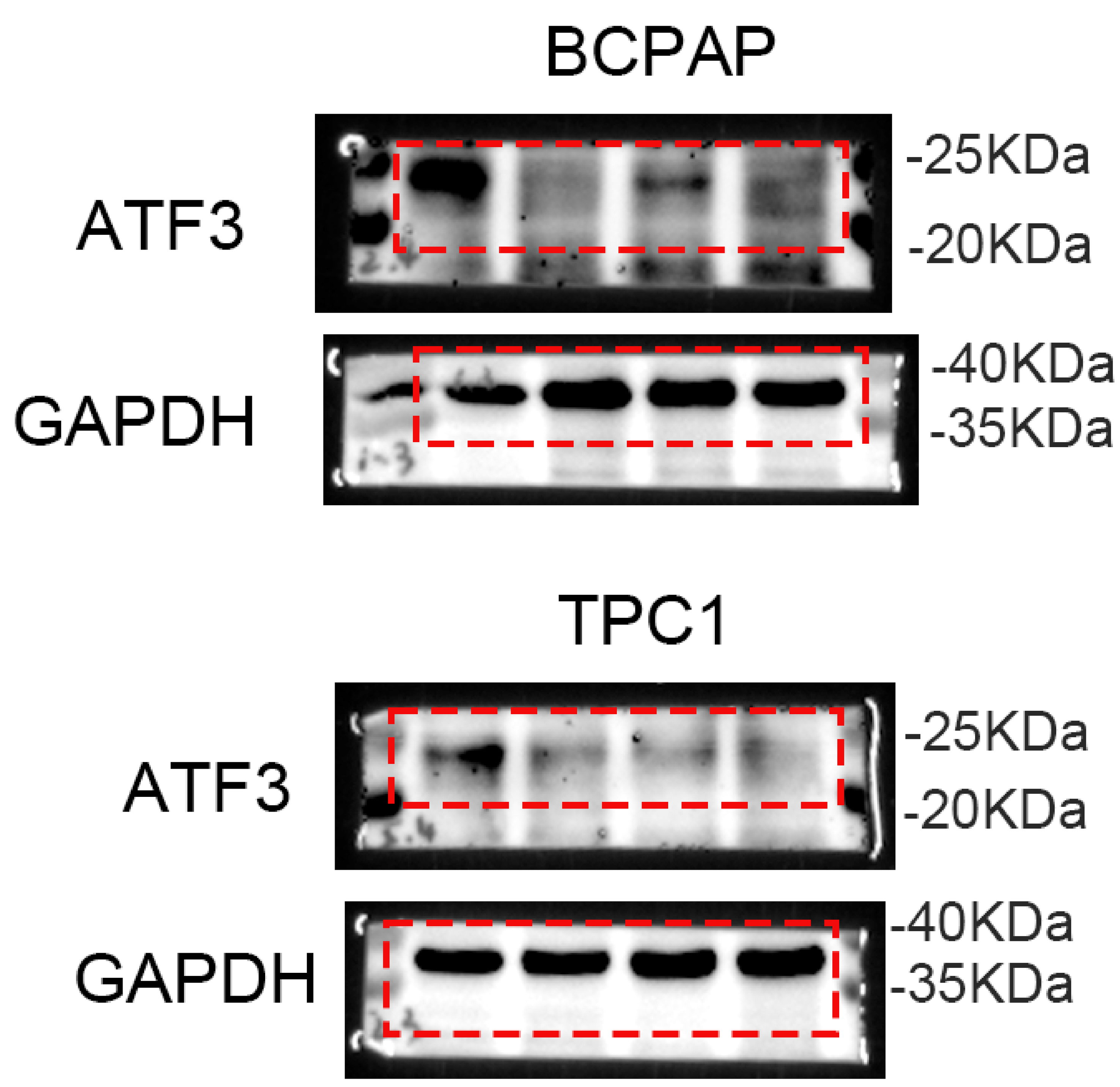

FigS6E

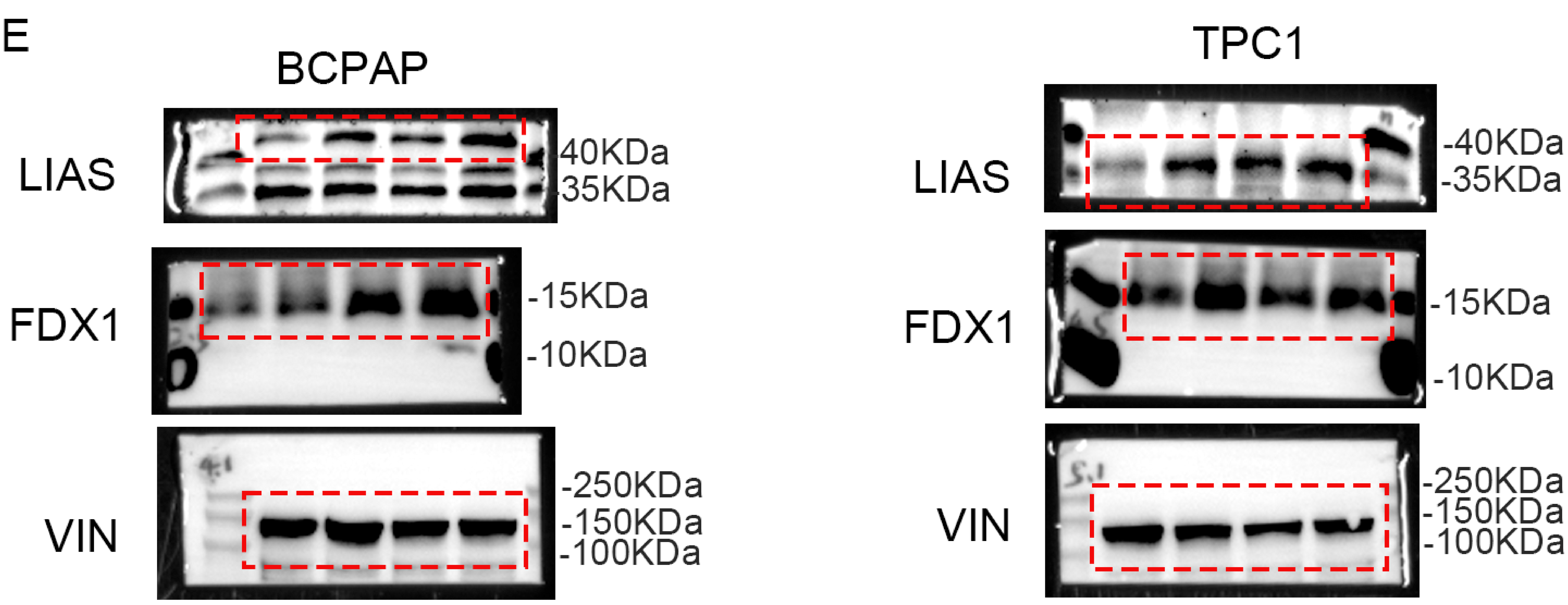

Supplement: Supplementary file 1 [file 41419_2026_8878_MOESM2_ESM.pdf]
