# Supplementary material for: Sunitinib enhances cuproptosis induced by copper ionophores via ROS-ATF3-SLC31A1 axis in thyroid Carcinoma
Source: Cell Death Dis. 2026 Jun 1;17(1):681. doi: 10.1038/s41419-026-08878-9 (PMC13434697; doi:10.1038/s41419-026-08878-9)
Supplement: Supplementary file 2 [file 41419_2026_8878_MOESM1_ESM.docx]

 Supplementary file for

Sunitinib enhances cuproptosis induced by copper ionophores via ROS-ATF3-SLC31A1 axis in thyroid Carcinoma

- **File list**
- Figure S1-S7
- Table S1-S3

**Supplementary Fig S1.** **Sunitinib exhibits synergistic antitumor activity with ES&Cu in vitro models.**

**A.** Pharmacological profiling of VEGFR2-targeting TKIs, Dose-response curves showing differential IC50 values in BCPAP and TPC1 cells (24-hour treatment). **B.** Synergistic response to the combination of Sunitinib and copper ionophore ES+Cu in TPC1 cell. The cells were fixed and stained after 10–14 days. Representative data from three independent experiments. **C.** Synergistic growth inhibition with ES-Cu combination therapy. Longitudinal cell viability assays demonstrated sustained suppression of PTC proliferation with sunitinib+ES-Cu co-treatment. Data are presented as mean ± standard deviation (SD). **D.** Synergistic growth suppression of B-CPAP and TPC-1 cells by Sunitinib and ES&Cu. Cell viability was assessed using combination treatments, followed by Chou-Talalay analysis. **E.** ES&Cu sensitivity across thyroid cancer subtypes (K1: papillary thyroid carcinoma [PTC]; TT/MZ: medullary thyroid carcinoma [MTC]; FTC-133: follicular thyroid carcinoma [FTC]; KHM-5M/SW1736/BHT-101/C-643: anaplastic thyroid carcinoma [ATC]) after Sunitinib treatment. Three independent experiments were performed. **P < 0.01, ***P < 0.001.

**Supplementary Fig S2. The combination of Sunitinib with ES&Cu simultaneously enhanced apoptosis and cuproptosis.**

**A.** Cell death inhibition assays using inhibitors targeting ferroptosis (ferrostatin-1, 5 μM), necroptosis (necrostatin-1, 10 μM), apoptosis (Z-VAD-FMK, 20 μM), and copper chelation (TTM, 10 μM) following ES&Cu treatment alone or Sunitinib/ES&Cu co-treatment. **B.** Flow cytometry quantification of apoptosis rates in BCPAP and TPC1 cells treated with ES&Cu alone or combined with Sunitinib (5 μmol/L, 24 h). Data are presented as mean ± standard deviation (SD). Three independent experiments were performed. **P < 0.01, ***P < 0.001.

**Supplementary Fig S3. The combination of Sunitinib with ES&Cu enhances cuproptosis.**

**A.**  Co-localization analysis of immunofluorescence (IF) imaging of DLAT aggregation (green) in B-CPAP and TPC1 cells treated for 24 h with: DMSO (control), 5 μM Sunitinib (Suni), 50 nM ES&Cu, Suni + ES&Cu (red, Mitotracker; blue, DAPI, Scale bars: 5 μm). **B.** Semi-quantitative outcomes derived from immunofluorescence analysis. **C.** Statistical analysis of Western blot data revealed significant reductions in expression of iron-sulfur cluster proteins (FDX1, DLAT, DLST, LIAS) and lipoylated DLAT following combination treatment with sunitinib and elesclomol-copper (ES+Cu). Data are presented as mean ± standard deviation (SD). Three independent experiments were performed. **P < 0.01, ***P < 0.001.

**Supplementary Fig S4. The combination of Sunitinib with ES&Cu significantly impaires cellular mitochondrial function.**

**A.** Statistical analysis of seahorse XF analysis demonstrated remarkable reduction in basal OCR with combination therapy vs monotherapy, indicating mitochondrial respiratory chain impairment. **B.** Statistical analysis of Western blot of OXPHOS showed Mitochondrial Complex II activity reduction following combination treatment. **C.** Mitochondrial gene expression signature. Heatmap of showed expression patterns genes related to lipoc acid pathway, PDH complex and Fe-S cluster with sunitinib treatment. Red: Significant genes. **D.**  ATP quantification revealed decrease in intracellular ATP levels with sunitinib+ES&Cu co-treatment (24h exposure, n=3). Data are presented as mean ± standard deviation (SD). Three independent experiments were performed. *P < 0.05, **P < 0.01, ***P < 0.001.

**Supplementary Fig S5.** **Sunitinib sensitizes PTC cells to ES&Cu-induced cuproptosis via SLC31A1 upregulation.**

**A.** Increased Fe-S cluster protein levels (FDX1, DLAT, LIAS) in SLC31A1-knockdowned TPC1 cells. **B.** Cell death inhibition assays using inhibitors targeting ferroptosis (ferrostatin-1, 5 μM), necroptosis (necrostatin-1, 10 μM), apoptosis (Z-VAD-FMK, 20 μM), and copper chelation (TTM, 10 μM) following Sunitinib/ES&Cu co-treatment before and after knockdown of SLC31A1. **C.** Co-localization analysis of immunofluorescence (IF) imaging of DLAT aggregation (green) in B-CPAP and TPC1 cells after overexpression of SLC31A1. **D.** Semi-quantitative outcomes derived from immunofluorescence analysis. Three independent experiments were performed. **P < 0.01.

**Supplementary Fig S6. Pan-cancer and thyroid-specific expression patterns of SLC31A1.**

**A.** Pan-cancer expression profiling. Analysis of TCGA data (n=10,535 samples across 33 cancer types revealed tissue-specific SLC31A1 expression). **B.** Thyroid carcinoma differential expression. TCGA data demonstrated significant SLC31A1 downregulation in PTC tumors vs unpaired or paired normal tissues. **C.** Reduced levels of SLC31A1 were observed in patients with stage T4 thyroid cancer. **D.** Decreased SLC31A1 was also associated with lymph node metastasis in thyroid carcinoma. **E.** SLC31A1 knockdown increased thyroid cell invasion in Matrigel Transwell assays (24h, 8μm pores). Data are presented as mean ± standard deviation (SD). Three independent experiments were performed. *P < 0.05, **P < 0.01, ***P < 0.001.

**Supplementary Fig S7.** **ATF3 regulates cuproptosis susceptibility.**

**A-B.** Pan-cancer expression profiling. Analysis of TCGA data (n=10,535 samples across 33 cancer types) revealed tissue-specific ATF3 expression. **C.** Statistical analysis of Western blot of ATF3 expression following Sunitinib exposure. **D.** Validation of ATF3 knockdown efficiency via shRNA in BCPAP and TPC1 cells, confirmed by Western blot. **E.**  Increased Fe-S cluster protein levels (FDX1, LIAS) in ATF3-knockdowned cells. **F.** Statistical analysis of ATF3 overexpression in BCPAP and TPC1 cells, confirmed by Western blot. **G.** IC50 shift quantification. ES&Cu IC50 decreased from 0.09μM to 0.05 μM with ATF3 overexpression (n=3; dose-response curve, four-parameter logistic fit). **H.** Semi-quantitative outcomes derived from immunofluorescence analysis. Data are presented as mean ± standard deviation (SD). **I.** Cell death inhibition assays using inhibitors targeting ferroptosis (ferrostatin-1, 5 μM), necroptosis (necrostatin-1, 10 μM), apoptosis (Z-VAD-FMK, 20 μM), and copper chelation (TTM, 10 μM) following Sunitinib/ES&Cu co-treatment before and after knockdown of ATF3. Three independent experiments were performed. *P < 0.05, **P < 0.01, ***P < 0.001.

**Table S1.** Sequences used for siRNAs in this study

| **Genes** | **Sequences (5’-3’)** |
| --- | --- |
| siNC | UUCUCCGAACGUGUCACGUTT  ACGUGACACGUUCGGAGAATT |
| siVEGFR2#1 | CGGGAAACUGACUUGGCCUCGTT  CGAGGCCAAGUCAGUUUCCCGTT |
| siVEGFR2#2 | GCGAUGGCCUCUUCUGUAAGATT  UCUUACAGAAGAGGCCAUCGCTT |

**Table S2.** Sequences used for shRNAs in this study

| **Genes** | **Sequences** |
| --- | --- |
| shSLC31A1#1 | GCATTCGCTACAATTCCATGC |
| shSLC31A1#2 | GCAACAGATGCTGAGCTTTCC |
| shSLC31A1#3 | GCGTAAGTCACAAGTCAGCAT |
| shATF3#1 | GGAAAGTGTGAATGCTGAACT |
| shATF3#2 | AAACAAGAAGGAGAAGA |
| shATF3#3 | CGAGAAGCAGCATTTGATATA |

**Table S3.** The primer sequences used for qPCR in this study

| **Genes** | **Primers sequences** |
| --- | --- |
| SLC31A1 | Forward: 5’- GGGGATGAGCTATATGGACTCC-3’ |
|  | Reverse: 5’- TCACCAAACCGGAAAACAGTAG -3’ |
| ATF3 | Forward: 5’-CTGGAAAGTGTGAATGCTGAAC-3’ |
|  | Reverse: 5’-ATTCTGAGCCCGGACAATAC-3’ |
| VEGFR2 | Forward:5’- GTGATCGGAAATGACACTGGAG-3’ |
|  | Reverse: 5’- CATGTTGGTCACTAACAGAAGCA-3’ |
| GAPDH | Forward: 5’-CAAGAGCACAAGAGGAAGAGAG-3’ |
|  | Reverse: 5’-CTACATGGCAACTGTGAGGAG-3’ |
| Chip site  (-94 to -81) | Forward: 5’-GTGGGGCAAAGGCAAATGAG-3’ |
|  | Reverse: 5’-TACCGACCAATCCCTGCAAG -3’ |
